# Dietary Fats, Serum Cholesterol and Liver Cancer Risk: A Systematic Review and Meta-Analysis of Prospective Studies

**DOI:** 10.3390/cancers13071580

**Published:** 2021-03-30

**Authors:** Longgang Zhao, Chuanjie Deng, Zijin Lin, Edward Giovannucci, Xuehong Zhang

**Affiliations:** 1 Department of Epidemiology & Biostatistics, Arnold School of Public Health, University of South Carolina, Columbia, SC 29208, USA; lz7@email.sc.edu; 2 Department of Epidemiology, Harvard T.H. Chan School of Public Health, Boston, MA 02115, USA; cdeng@hsph.harvard.edu (C.D.); egiovann@hsph.harvard.edu (E.G.); 3 Beth Israel Deaconess Medical Center, Harvard Medical School, Boston, MA 02115, USA; annielin0926@gmail.com; 4 Department of Nutrition, Harvard T.H. Chan School of Public Health, Boston, MA 02115, USA; 5 Channing Division of Network Medicine, Department of Medicine, Brigham and Women’s Hospital and Harvard Medical School, Boston, MA 02115, USA

**Keywords:** meta-analysis, liver cancer, dietary fat, cholesterol, high-density lipoprotein cholesterol, low-density lipoprotein cholesterol, epidemiological study

## Abstract

**Simple Summary:**

Due to the rapid increase of primary liver cancer incidence and the poor prognosis, it is imperative to identify new modifiable factors such as diet and nutrition for the prevention of liver cancer. Diet high in saturated fatty acids (SFA) has been hypothesized to be associated with increased risk of cancers. However, the associations between dietary fatty acids and liver cancer are not consistent. We aimed to examine the association between dietary total fat, its major components, serum cholesterol, and risk of liver cancer combining current evidence from prospective studies. Our meta-analyses provided new evidence on associations between dietary fats, serum cholesterol, and liver cancer risk. Higher intake of dietary SFA was associated with higher risk of liver cancer while higher serum levels of cholesterol and high-density lipoprotein (HDL) were associated with a lower risk of liver cancer with high between-studies variability. Based on our findings, reducing dietary SFA may help to prevent the development of liver cancer.

**Abstract:**

To quantify the associations between dietary fats and their major components, as well as serum levels of cholesterol, and liver cancer risk, we performed a systematic review and meta-analysis of prospective studies. We searched PubMed, Embase, and Web of Science up to October 2020 for prospective studies that reported the risk estimates of dietary fats and serum cholesterol for liver cancer risk. We carried out highest versus lowest intake or level and dose-response analyses. Higher intake of dietary saturated fatty acids (SFA) was associated with a higher liver cancer risk in both category analysis (relative risk [RR]_highest vs. lowest intake_ = 1.34, 95% confidence interval [CI]: 1.06, 1.69) and dose-response analysis (RR_1% energy_ = 1.04, 95%CI: 1.01, 1.07). Higher serum total cholesterol was inversely associated with liver cancer but with large between-studies variability (RR_1 mmol/L_ = 0.72, 95%CI: 0.69, 0.75, I^2^ = 75.3%). The inverse association was more pronounced for serum high-density lipoprotein (HDL) cholesterol (RR_1 mmol/L_ = 0.42, 95%CI: 0.27, 0.64). Higher intake of dietary SFA was associated with higher risk of liver cancer while higher serum levels of cholesterol and HDL were associated with a lower risk of liver cancer with high between-studies variability.

## 1. Introduction

Primary liver cancer is the sixth most frequently diagnosed cancer and the fourth leading cause of cancer-related death worldwide in 2018 [1]. Current known risk factors for liver cancer include chronic infections (e.g., hepatitis B virus, hepatitis C virus), metabolic diseases (e.g., type 2 diabetes, obesity), behavioral factors (e.g., alcohol consumption, tobacco), and aflatoxin-contaminated foods [2]. In the past several decades, we have witnessed an increase in the incidence of liver cancers in Western countries [3]. However, these established risk factors combined can only explain less than 60% of all liver cancers in the U.S. [4]. Therefore, it is imperative to identify other modifiable factors, such as diet and nutrition.

The liver is the main organ for the synthesis and circulation of fatty acids and cholesterol in the human body. The relationship between dietary fat intake and liver cancer risk has been long of interest to researchers. Experimental and animal studies from the last several decades have demonstrated that high-fat diets can increase the risk of steatohepatitis and liver cancer in mice via the accumulation of fat and cholesterol [5]. However, limited prospective studies investigated the associations between dietary fat and liver cancer risk and reported inconsistent results [6,7,8]. In addition, any influence on the development of liver cancer may depend on the types of fatty acids. Due to the lower power of individual studies with a limited number of liver cancer cases, combining all the available evidence using meta-analysis methods will enhance the power to detect significant associations with liver cancer not only for dietary fats but also for different types of fatty acids.

A high blood level of total cholesterol, which could be affected by dietary saturated fatty acids (SFA), is a well-established risk factor for coronary heart disease and stroke [9]. However, the relationship between total cholesterol and the risk of cancer remains uncertain [10]. The different types of serum cholesterol, the high-density lipoprotein (HDL) and low-density lipoprotein (LDL) cholesterol, may play different roles in etiology of liver cancer according to their functions in cholesterol metabolism. Recently, several studies showed the associations between serum cholesterol and liver cancer risk in different populations and reported inverse associations but with different magnitudes [11,12].

Hence, we examined the association between dietary total fat and its major components including SFA, monounsaturated fatty acids (MUFA), N-3 and N-6 polyunsaturated fatty acids (PUFA), serum cholesterol including HDL and LDL cholesterol, and risk of liver cancer combining current evidence from prospective studies. We hypothesized that dietary SFA, but not MUFA and PUFA would be associated higher risk of liver cancer. We also hypothesized that higher level of cholesterol, if driven by HDL rather than LDL cholesterol, would be inversely associated with liver cancer risk.

## 2. Materials and Methods

The current review was conducted and reported in accordance with the standard criteria (Preferred Reporting Items for Systematic Reviews and Meta-Analyses, PRISMA) [13].

### 2.1. Search Strategy

We searched PubMed, Embase, and Web of Science up to October 2020. Details of the search terms are provided in Table S1. We did not apply any year, language, or publication status restrictions to the selection of articles for inclusion. We also searched the reference of the retrieved studies for any additional studies.

### 2.2. Study Selection and Inclusion Criteria

Two researchers (C.D. and Z.L.) independently performed the study selection. First, we scanned the title and abstract to obtain the relevant literature for further full-text review. Second, we downloaded the identified literature and read the full text carefully to ascertain the target literature through our inclusion criteria. The inclusion criteria included as follows: (1) the exposure of interest was dietary fat intake or its subtypes or serum cholesterol level; and (2) the outcome of interest was incidence of primary liver cancer or hepatocellular carcinoma or intrahepatic bile duct carcinoma; and (3) the study design was cohort design or nested case-control design, or case-cohort design; and (4) the study reported adjusted relative risk (RR) estimates and 95% confidence intervals (CI). An exclusion list of full-text review was provided in Table S2.

### 2.3. Data Extraction and Quality Assessment

Two researchers (C.D. and Z.L.) performed the data extraction independently and the discrepancy was solved with another researcher (L.Z.). We used a priori abstract table to obtain the information including last name of first author, publication year, country, name of the study, follow-up period, cohort size or numbers of participants, liver cancer cases, sex, baseline age, population exclusion criteria, type of outcome and its ascertainment, type of fat or serum cholesterol and its assessment, amount or frequency of exposures, RRs and 95%CI, variables adjusted for in the analysis.

We used the Newcastle–Ottawa scale (NOS) to assess the risk of bias of the included studies based on selection bias, comparability, and outcome assessment [14]. We considered studies with 0–3, 4–6, and 7–9 points to represent low-, medium-, and high-quality studies, respectively (Table S3).

### 2.4. Patient Involvement

No patients were involved in setting the research question or the outcome measures, nor were they involved in developing plans for design or implementation of the study. No patients were asked to advise on interpretation or writing up of results.

### 2.5. Statistical Methods

#### 2.5.1. Statistical Synthesis

We conducted two types of analyses: associations for the highest versus lowest categories and dose-response analyses using the random effects model by DerSimonian and Laird, which considers variation both within and between studies [15]. For dose-response analysis, we estimated the RR for one unit of exposure as follows: for serum cholesterol, HDL, LDL, per 1 mmol/L; for dietary cholesterol, per 100 mg/day; for total fat, per 5% of energy from total fat; for subtypes of fatty acids, per 1% of energy from a specific type of fat; for N-6/N-3 PUFA ratio, per 1 unit change; for other fat ratios, per 0.1-unit change. We both estimated the RR and 95%CI for each increment of per one-unit increase and explored the nonlinear associations if appropriate. For each study, the trend from the correlated log RRs across categories of fat intake or cholesterol level was calculated using the method proposed by Greenland et. al. [16]. We assigned the midpoint of fat intake or cholesterol level of each category to the corresponding risk estimates of each study. If the upper bound in the highest category was not available, we assumed that it had the same amplitude as the preceding one. For nonlinear associations, we applied a two-stage, random-effect dose-response meta-analysis by modeling fat intake or cholesterol level using restricted cubic splines with three knots at fixed percentiles (5%, 50%, and 95%) of the distribution. We first fitted a restricted cubic spline model into each set of RRs within a specific study and then combined the two regression coefficients and the variance/covariance matrices for each study using a multivariate random-effects model. *p* value for nonlinearity was calculated by testing whether the coefficient of the second spline was equal to zero [17].

#### 2.5.2. Heterogeneity

We evaluated heterogeneity by estimating the variance between studies using Cochran’s Q test and the I-squared (I^2^) statistic. We used *p* < 0.10 for Q test or I^2^ > 50% as statistically significant. I^2^ is the amount of total variation explained by variation between studies [18]. We did not conduct meta-regression analyses because of the limited number of studies for each exposure.

#### 2.5.3. Sensitivity Analyses and Publication Bias

Influential analyses by excluding one study at a time from each analysis were conducted to investigate the robustness of the findings. Indication of small study effects was evaluated based on Egger’s regression asymmetry test (*p* = 0.10) [19]. If there was evidence of publication bias, we used the trim-fill methods to reanalyze the data.

#### 2.5.4. Software Used

All statistical analyses were performed using the R program (Version 3.5.0, R core team, Vienna, Austria). A two-sided *p* value less than 0.05 was considered as statistical significance if not specified.

## 3. Results

### 3.1. Literature Research and Data Abstraction

**Figure 1:**
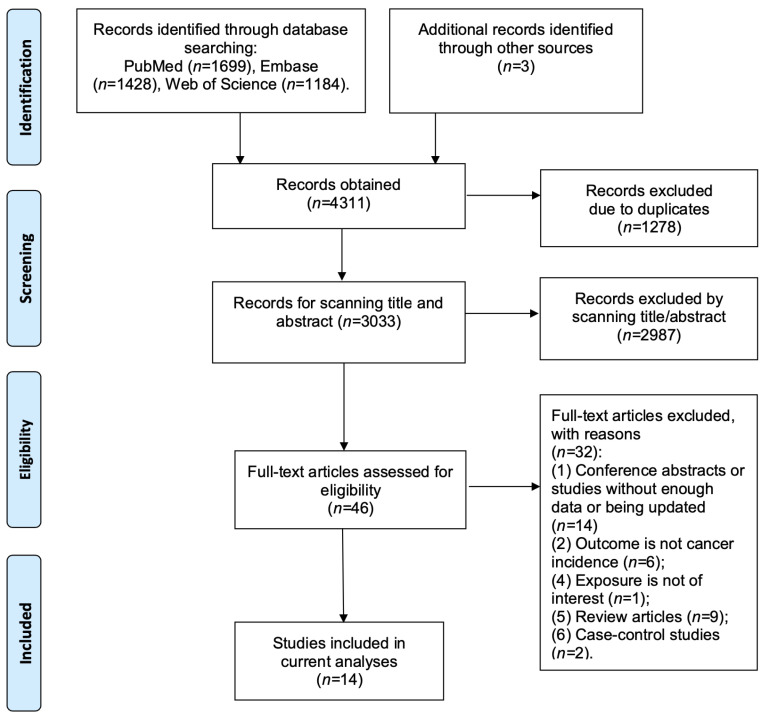
Follow chart of study selection.

We identified 3033 records by searching PubMed, Embase, and web of science after removing the duplicates (Figure 1). We identified 46 articles that needed further full-text review, of which 32 articles were excluded (Table S2). Finally, we included 15,890 liver cancer cases from 14 prospective studies in the current meta-analysis [6,7,8,11,12,20,21,22,23,24,25,26,27,28] (Table 1).

### 3.2. Dietary Fats and Liver Cancer Risk

**Figure 2:**
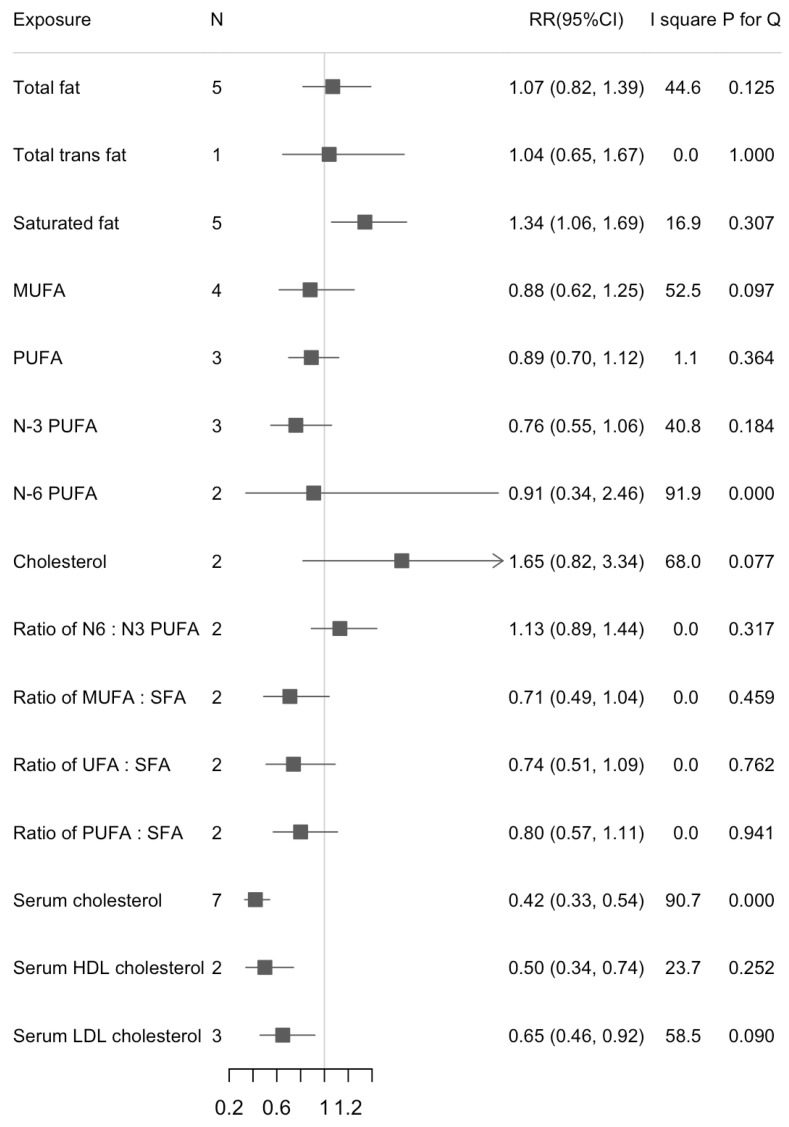
Summary of associations between dietary fats and serum cholesterol and liver cancer (Highest versus lowest categories).

**Figure 3:**
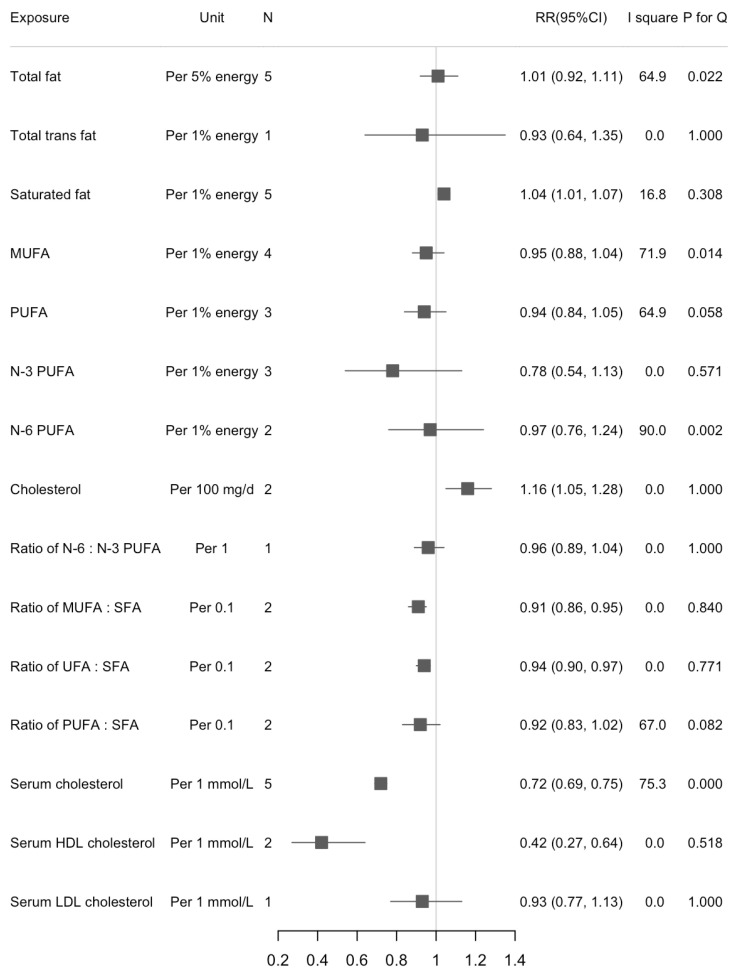
Summary of associations between dietary fats and serum cholesterol and liver cancer (dose-response analysis).

We found a statistically significant association between dietary SFA and liver cancer risk in the highest versus lowest intake (RR = 1.34, 95%CI: 1.06, 1.69; *n* = 5, I^2^ = 16.9%) (Figure 2). The increased risk of liver cancer with higher dietary SFA was also found in the dose-response analysis (RR_1% energy increase_ = 1.04, 95%CI: 1.01, 1.07; *n* = 5, I^2^ = 16.8%) (Figure 3). There were statistically inverse associations between per 0.1-unit increase in ratio of MUFA:SFA, unsaturated fatty acids (UFA):SFA, and liver cancer risk with RRs (95%CIs) of 0.91 (0.86, 0.95), and 0.94 (0.90, 0.97), respectively. An inverse association between dietary cholesterol intake and liver cancer risk was found in dose-response analyses but not in highest versus lowest analyses (RR_100 mg/d_ = 1.16, 95%CI: 1.05, 1.28; *n* = 2, I^2^ = 0%). We did not find any significant associations between intake of dietary total fat, MUFA, and PUFA and risk of liver cancer. Results for highest versus lowest categories and dose-response analyses were provided in Figure 2 and Figure 3, respectively. The forest plots for the above associations were shown in Figures S5–S10, S13–S18. We did not detect any nonlinear associations between dietary total fat, SFA and liver cancer risk (Figures S1 and S2).

### 3.3. Serum Cholesterol and Liver Cancer Risk

We found an inverse association between serum total cholesterol and liver cancer risk when comparing the highest versus lowest categories (RR = 0.42, 95%CI: 0.33, 0.54; *n* = 7, I^2^ = 90.7%) (Figure 2). Results were similar in separate analyses for men (RR_H/L_ = 0.39, 95%CI: 0.27, 0.57) and women (RR_H/L_ = 0.31, 95%CI: 0.26, 0.38). For the subtypes of cholesterol, serum HDL cholesterol appeared more strongly associated with liver cancer risk than serum LDL cholesterol (RR_HDL_ = 0.50, 95%CI: 0.34, 0.74; *n* = 2, I^2^ = 23.7%; RR_LDL_ = 0.65, 95%CI: 0.46, 0.92; *n* = 3, I^2^ = 58.5%). When we calculated the RRs for per 1 mmol/L increase in serum cholesterol, the associations were stable except for serum LDL cholesterol. Serum cholesterol was inversely associated with risk of liver cancer but with large between-study heterogeneity (RR_1 mmol/L_ = 0.72, 95%CI: 0.69, 0.75; *n* = 7, I^2^ = 75.3%) (Figure 3). The RR decreased 58% for per 1 mmol/L increase of HDL cholesterol (RR_1 mmol/L_ = 0.42, 95%CI: 0.27, 0.64; *n* = 2, I^2^ = 0.0%). Results were provided in Figure 2 and Figure 3. The forest plots for above associations were shown in Figures S4, S11 and S12. A significant nonlinearity was found between serum cholesterol and liver cancer risk, which indicates a L-curve with an inflection at about 6 mmol/L (*p* for nonlinearity < 0.001, Figure S3).

### 3.4. Publication Bias

We performed tests for small study effect and sensitivity analyses when the number of studies in each association was larger than six. We found the association between serum cholesterol and liver cancer risk might be affected by publication bias. The *p* values for Egger’s test from highest versus lowest categories and per 1 mmol/L increase of serum cholesterol were 0.064 and 0.005. Therefore, we used the trim-fill methods to adjust the publication bias. However, the results did not change materially compared with the main results (Figure S19a,b).

We also performed sensitivity analyses to explore the robustness of our main findings. We used the leave-one-out methods and found the main findings were stable and robust in general when we excluded one study one time (Table S4). When we excluded studies with a NOS less than seven (only one study), the results did not change significantly (data not shown). We also conducted analyses by excluding Li et al. [12] study due to its short follow-up time and found association between LDL and liver cancer became statistically nonsignificant (RR_H/L_ = 0.78, 95%CI: 0.59–1.03, I^2^ = 0.0%).

## 4. Discussion

### 4.1. Principal Findings

For the first time, our study provided a comprehensive analysis synthesizing evidence on dietary fat intake, serum cholesterol, and liver cancer risk. We found an increased risk for dietary SFA with liver cancer using both category and dose-response analyses. Higher ratios of MUFA:SFA and UFA:SFA were associated with a lower risk of developing liver cancer. Higher serum cholesterol and HDL were associated with a lower risk of liver cancer with high between-studies variability. These findings were generally robust and stable in sensitivity analyses.

The associations between dietary fats and health outcomes have been a long-standing research topic of interest [29]. Although not entirely consistent, some evidence suggests that specific types of fat may play different roles in carcinogenesis, and replacing saturated fats with unsaturated fats may be beneficial to the prevention of some specific cancers including breast cancer [30], pancreatic cancer [31], prostate cancer [32], and lung cancer [33], but not colorectal cancer [34,35]. In general, our results provided new evidence for liver cancer by adding that higher intake of dietary SFA was associated with an increased risk of liver cancer. Additionally, we found inverse associations between ratio of MUFA:SFA, UFA:SFA, and liver cancer risk. However, due to the limited number of studies, these results should be interpreted with caution.

The positive association between dietary SFA and liver cancer risk is biologically plausible. In experimental studies, excess ingestion of SFA increases hepatic lipid storage, energy metabolism, and insulin resistance [36], which may directly contribute to development of nonalcoholic fatty liver disease (NAFLD), a risk factor for liver cancer [37]. Dietary nutrients such as fats and sugar are involved in the development of hepatic steatosis [38]. In addition, evidence has accumulated that higher dietary SFA was also associated with increased risk of type 2 diabetes and obesity [39,40], risk factors for liver cancer. When we evaluated the association between SFA and liver cancer, four [6,7,8,24] of five studies adjusted for total energy intake while the remaining one [22] additionally adjusted for other fatty acids types. A combination of studies replacing SFA with carbohydrate or protein or studies replacing SFA with all other types of macronutrients except for SFA yielded similar results (data not shown).

A large body of evidence indicates that higher intake of dietary SFAs increases blood levels of LDL cholesterol and the LDL to HDL ratio, both of which are associated with a higher risk of cardiovascular diseases [41]. The epidemiological evidence on associations between serum cholesterol and cancer risk has accumulated in recent decades [9,10,11,12]. Cholesterol is a unique lipid, essential for membrane biogenesis, cell proliferation, and cell differentiation. It is not only from the diet but is also mainly synthesized by the liver in humans and distributed throughout the body via LDL and HDL transporters. Previous studies indicate that serum cholesterol is positively associated with the risk of some specific cancers because it is the obligatory precursor of steroid hormones involved in tumor promotion and tumor death [10]. The positive associations of serum cholesterol were shown for risk of breast and prostate cancer but not for all cancer types [11].

In the current analysis, we found an inverse association between serum cholesterol and liver cancer risk although higher between-study variability was observed. The underlying mechanisms are largely unclear. First, unmeasured factors that could decrease the serum cholesterol level and lead to the development of liver cancer may be possible, such as unknown genotypes. For example, transmembrane 6 superfamily 2 (TM6SF2) rs5854292-T variant is a risk factor for NAFLD and liver cancer [42,43]. In animal studies, TM6DF2 rs5854292-T is associated with lower serum levels of total cholesterol and LDL [42]. Thus, some genetic susceptibility may influence the association between serum cholesterol and liver cancer, which requires further investigation. Other mutations such as rs738409-G (PNPLA3) and rs1800562-A (HFE) [44] could also influence the observed association between serum cholesterol and liver cancer. Second, undiagnosed liver diseases, such as NAFLD and cirrhosis, could influence cholesterol metabolism, possibly exaggerating the inverse association with liver cancer risk. Additionally, chronic hepatitis B virus infection is associated with both higher liver cancer risk and lower cholesterol concentration, which may provide qualitative confounding between blood cholesterol level and liver cancer [45]. In this context, the higher levels of cholesterol might be an indicator of liver function, rather than a causal factor for liver cancer risk. Among 14 studies we included in this meta-analysis, only 3 studies excluded participants who had cirrhosis or other liver diseases. Therefore, we cannot fully evaluate the impact of undiagnosed liver diseases on our results. Third, metabolic disorders are risk factors for liver cancer and are also associated with abnormal serum cholesterol level. It may have influenced the associations between serum cholesterol, especially HDL, and liver cancer risk [46]. Fourth, it is also possible that this association may be due to increased use of cholesterol-lowering drugs such as statin in patients with elevated cholesterol [47]. Stain use has been reported to be associated with a lower risk of liver cancer [48]. We could not fully evaluate the influence of statin use on our results because most studies on serum cholesterol-liver cancer have not adjusted for statin use. Nonetheless, statin use was not common before 1990 [49]. We thereby restricted our analyses for studies with a baseline survey before 1990 and found an inverse association between serum cholesterol level and liver cancer risk (OR_H/L_ = 0.60, 95%CI: 0.46, 0.78, *n* = 3, I^2^ = 81.2%). Clearly, future studies with statin use data are warranted.

### 4.2. Implications

Our meta-analysis provides new evidence on associations between dietary fats, serum cholesterol, HDL, and liver cancer risk. Previous studies mainly elucidate the detrimental effect of dietary SFA intake and the benefits of replacing SFA with unsaturated fatty acids for the prevention of cardiovascular diseases. Based on our findings, reducing dietary SFA may also help to prevent the development of liver cancer. However, for other subtypes of fats such as N-3 and N-6 PUFA, more studies are needed because the current studies are limited. The inverse association between serum cholesterol and liver cancer is unexpected, which requires further investigation.

### 4.3. Strengths and Limitations

Our study has several strengths. First, we provided estimates for both category analysis and dose-response analysis. We also explored the potential nonlinearity between dietary fats, serum cholesterol, and liver cancer risk. Second, we only included prospective studies, which may increase the internal validity of our findings. Third, for most serum cholesterol studies (7 of 8 studies), the blood samples were collected at the cohort baseline and the follow-up time are over 10 years, which possibly minimized the potential concerns of reverse causation.

Several limitations also merit attention. First, the number of existing studies is relatively small, which led to lower statistical power to detect the associations and publication bias. For some associations such as the ratio of different subtypes of fatty acids, only one or two studies were retrieved. Second, our study is also subject to the limitations that affected the original studies. For example, dietary information in the included studies was mostly collected by FFQs, which may lead to measurement errors. Third, the information for HBV and HCV is not available in most studies. Therefore, we cannot evaluate the influence of HBV or HCV status on our results. However, the HBV/HCV seems unlikely to substantially confound the reported associations between diet and liver cancer [50]. Fourth, there is substantial between-study heterogeneity between serum cholesterol and liver cancer. We did not conduct meta-regression to explore the source of the heterogeneity due to the limited number of studies. Our findings also indicate that there is publication bias in associations between serum cholesterol and liver cancer. However, after applying a trim-filled method to adjust for the publication bias, our findings did not materially change. Lastly, in the current meta-analysis, 7 out of 8 studies for serum cholesterol used liver cancer as a main outcome and 5 out of 6 studies for dietary fat focused on HCC risk. Thus, our results apply largely to HCC, and we cannot fully evaluate the potential etiology heterogeneity by different subtypes of liver cancer.

## 5. Conclusions

Our meta-analysis supported that avoiding excess dietary SFA intake would be beneficial to the prevention of liver cancer. Higher ratio of MUFA:SFA and PUFA:SFA were associated with a lower risk of developing liver cancer. We also found higher serum levels of cholesterol and HDL cholesterol were associated with a lower risk of liver cancer with high between-study heterogeneity. The underlying mechanisms need to be further investigated.

## Figures and Tables

**Table 1:** Characteristics of prospective cohort studies included in meta-analysis on associations between dietary fats and serum cholesterol and liver cancer.

Author, Year (Reference)	Country, Study Design	Baseline Years	Median Follow-Up (Years)	Age (Mean/Median, Years)	Sex	Population Exclusion	Cohort Size	Cases	Exposure	Exposure Assessment	Outcome	Outcome Assessment	Adjustment for Confounding Variables	NOS Score
Li, 2020 [12]	China, 4C Cohort	2010–2014	3.8	56.9	Both	Participants diagnosed as cancer within 6 months from baseline were excluded.	137,884	156	LDL cholesterol	Serum level	Liver cancer	Medical records	Age, sex, BMI, family history of cancer, smoking, drinking, education status, physical activity, consumption of vegetables and fruit, insulin therapy, lipid-lowering medication, and systolic blood pressure.	8
Yang, 2020 [8]	U.S., NHS/HPFS	1980	26.6	Median = 64–68	Both	Cancer diagnosed before baseline except for non-melanoma skin cancer were excluded.	138,483	160	Total fat, SFA, MUFA, PUFA, Trans fat, cholesterol, N3 PUFA, N6 PUFA, N6/N3 ratio, P:S ratio, M:S ratio, (M + P):S ratio	Validated FFQ	HCC	Biennial questionnaires, medical records and pathological reports confirmed by a study physician, state cancer registries, the National Death Index, death certificate	Age, sex, race, physical activity, BMI, smoking status, aspirin use, type 2 diabetes, alcohol intake, total coffee intake, and total energy intake.	8
Nderitu, 2017 [26]	Sweden, Swedish AMORIS	1985	20.03	44	Both	Participants with benign liver tumors, primary liver cancer or cirrhosis at baseline were excluded.	509,436	766	Cholesterol, HDL cholesterol, LDL cholesterol	Serum level	Liver cancer	Linkage with Swedish national registries	Age, sex, SES, triglycerides, cholesterol, raised glucose, diabetic status and history of liver disease (Cholesterol not adjusted for total cholesterol; HDL-C, LDL-C not adjusted for triglycerides).	7
Guan, 2017 [23]	China, Kailuan Cohort Study	2006	approximately 8 years	51.05	Male	Participants with history of cancer and cardiovascular disease were excluded.	68,759	205	Cholesterol, LDL cholesterol	Serum level	Liver cancer	Medical insurance, hospital records/death confirmed by death certificates	Age, cigarette smoking, alcohol consumption, physical activity, hypertension, diabetes mellitus, BMI.	7
Koh, 2016 [7]	Singapore, SCHS	1993	14	56.4	Both	Individuals who had history of invasive cancer at baseline (except non-melanoma skin cancer) were excluded.	60,298	488	Total fat, SFA, MUFA, N3 PUFA, N6 PUFA, N6/N3 ratio	Validated FFQ	HCC	Record linkage analysis of the cohort database with databases of the population-based Singapore Cancer Registry and Singapore Registry of Births and Deaths	Age, sex, dialect, year of interview, educational level, BMI, smoking status, alcohol use, coffee drinking status, baseline history of self-reported diabetes, total energy and dietary protein. Fat subtype intakes are mutually adjusted.	9
Duarte-Salles, 2015 [6]	Europe, EPIC	1992	11.4	51.2	Both	Generally healthy population across centers.	477,206	191	Total fat, SFA, MUFA, PUFA, P:S ratio, M:S ratio, (P + M):S ratio	Validated FFQ and 24-h dietary recall	HCC	Record linkage with cancer registries/a combination of methods.	Baseline alcohol intake and non-alcohol total energy intake, sex-specific physical activity level, BMI, smoking status, lifetime alcohol intake pattern, coffee intake, and intake of dietary fiber. Fat subtype intakes are mutually adjusted.	9
Sawada, 2012 [27]	Japan, JPHC	1990	11.2	40–69	Both	Subjects who had been diagnosed with cancer before the starting point were excluded.	90,296	398	N3 PUFA	Validated FFQ	HCC	Data linkage of major local hospitals with cancer registries/death certificate	Age, area, sex, smoking status, alcohol frequency, BMI, past history of diabetes mellitus, intake of coffee, soy foods, vegetables, vegetable oil, protein, and iron.	9
Borena, 2011 [21]	Europe, Me-Can cohort	2006	12	44	Both	Malignant cancer before the health examination were excluded.	578,700	266	Cholesterol	Serum level	Liver Cancer	National cancer registries	Age, smoking status, cohort, birth year and sex, BMI.	8
Kitahara, 2011 [11]	Korea, KCPS	1992–1995	12.7	44.9 (men), 49.3 (women)	Both	Participants who reported having cardiovascular disease, cancer, liver disease, or a respiratory disease at or before the initial visit, or who had extremely low levels of BMI, with missing or implausibly high or low total cholesterol levels were excluded.	1,189,719	10,161	Cholesterol	Serum level	Liver cancer	Medical records	Cigarette smoking, alcohol drinking, BMI, fasting serum glucose, hypertension, and physical activity.	9
Freedman, 2010 [22]	U.S., NIH-AARP	1995	NR	Median = 62.6	Both	Participants who developed cancer or died before their questionnaires were scanned were excluded.	495,006	338	Total fat, SFA, MUFA, PUFA	Validated FFQ	HCC	State cancer registries	Age, sex, alcohol, BMI, cigarette smoking, diabetes, education, fruit intake, vegetable intake, marital status, race and/or ethnicity, total energy from nonalcohol sources, usual physical activity throughout the day, and vigorous physical activity.	7
Ahn, 2009 [20]	U.S., ATBC	1985	14.9	50–69	Male	Participants with history of cancer other than nonmelanoma skin cancer or carcinoma in situ, severe angina pectoris, chronic renal insufficiency, liver cirrhosis, chronic alcoholism, anticoagulant therapy, other medical problems that might have limited long-term participation were excluded.	29,093	191	Cholesterol, HDL cholesterol	Serum level	Liver cancer	Medical records	Age, intervention, level of education, systolic blood pressure, BMI, physical activity, duration of smoking, number of cigarettes smoked per day, saturates fat intake, polyunsaturated fat intake, total calorie, alcohol consumption, and serum HDL cholesterol.	9
Ioannou, 2009 [24]	U.S., NHANES	1971	13.3	48.8	Both	Participants who reported at baseline ever being told by a physician that they had jaundice, hepatitis, or a malignant tumor, who had hepatomegaly or splenomegaly at baseline examination, or whose level of serum albumin was less than 3 g/dL were excluded.	9221	123	Total fat, SFA, cholesterol	24-h dietary recall questionnaire	Cirrhosis and liver cancer	Hospitalization records death certificates	Energy from other macronutrients, daily alcohol consumption, coffee or tea, gender, race, age, education, region, diabetes, BMI, and subscapular-to-triceps skinfold ratio.	6
Iso, 2009 [25]	Japan, JPHC	1990	12.4	40–69	Both	Participants with history of cardiovascular disease were excluded.	33,368	125	Cholesterol	Serum level	Liver cancer	Medical records and/or cancer registries	Age, BMI, pack years of smoking, ethanol intake, hypertension, diabetes, hyperlipidemia medication use, total vegetable intake, coffee intake and public health center.	9
Strasak, 2009 [28]	Australia, VHM and PP	1985	11.6	41.6	Both	Healthy participants, free of cancer at baseline.	172,210	2322	Cholesterol	Fasting blood sample	Malignant neoplasms of digestive organs	Vorarlberg Cancer Registry	Age, BMI, smoking status, occupational status, and year of entry into the cohort.	8

Abbreviations: 4C, the China Cardiometabolic Disease and Cancer Cohort Study; AMORIS, The Swedish Apolipoprotein Mortality Risk Study; ATBC, the Alpha-Tocopherol, Beta-Carotene Cancer Prevention Study cohort; BMI, body mass index; EPIC, the European Prospective Investigation into Cancer and Nutrition cohort; FFQ, food frequency questionnaire; H-B, Hospital based; HCC, hepatocellular carcinoma; HDL, high-density lipoproteins; HPFS, the Health Professional Follow-up Study; JPHC, the Japan Public Health Center-based prospective study; KCPS, the Korean Cancer Prevention Study; LDL, low-density lipoproteins; M:S ratio, the ratio of MUFA and SFA; (M + P):S ratio, the ratio of unsaturated fat and saturated fat; MUFA, monounsaturated fatty acids; NHANES, the National Health and Nutrition Examination Survey; NHS, the Nurse Health Study; NOS, Newcastle-Ottawa Quality Assessment Scale; NR, not reported; SCHS, the Singapore Chinese Health Study; SES, social economic status; SFA, saturated fatty acids; PUFA, polyunsaturated fatty acids; P:S ratio, the ratio of PUFA and SFA; VHM and PP, the Vorarlberg Health Monitoring and Promotion Program.

## Data Availability

The full dataset and statistical code are available from the corresponding author.

## Supplementary Materials

The following are available online at https://www.mdpi.com/article/10.3390/cancers13071580/s1, Table S1: Search terms in current meta-analysis, Table S2: Studies reviewed in full text for eligibility (excluded reasons for meta-analysis), Table S3: Quality assessment of studies included in meta-analysis (Newcastle-Ottawa Quality Assessment Scale), Table S4: Sensitivity analyses of associations between dietary fat, serum cholesterol and liver cancer (number of studies ≥ 6), and Figure S1: Association between serum cholesterol and liver cancer, Figure S2: Association between total dietary fat and liver cancer, Figure S3: Association between dietary saturated fat and liver cancer, Figures S4–S10: Forest plots of associations between dietary fats and serum cholesterol and liver cancer (per 1-unit increase), Figures S11–S18: Forest plots of associations between dietary fats and serum cholesterol and liver cancer (Highest vs. Lowest categories), Figure S19: Trim-fill methods to adjust publication bias for associations indicating potential publication bias.
