# Supplementary material for: Dietary Fats, Serum Cholesterol and Liver Cancer Risk: A Systematic Review and Meta-Analysis of Prospective Studies
Source: Cancers (Basel). 2021 Mar 30;13(7):1580. doi: 10.3390/cancers13071580 (PMC8037522; doi:10.3390/cancers13071580)

**Dietary Fats, Serum Cholesterol and Liver Cancer Risk: A Systematic Review and Meta-analysis of Prospective Studies**

**Author:** Longgang Zhao, Chuanjie Deng, Zijin Lin, Edward Giovannucci, Xuehong Zhang

**Appendix materials**

*Appendix 1: Supplementary tables*

Table S1. Search terms in current meta-analysis
Table S2. Studies reviewed in full text for eligibility (excluded reasons for meta-analysis)
Table S3. Quality assessment of studies included in meta-analysis (Newcastle-Ottawa Quality Assessment Scale)
Table S4. Sensitivity analyses of associations between dietary fat, serum cholesterol and liver cancer (number of studies ≥ 6)

Figure S1. Association between serum cholesterol and liver cancer
Figure S2. Association between total dietary fat and liver cancer
Figure S3. Association between dietary saturated fat and liver cancer
Figure S4-S10. Forest plots of associations between dietary fats and serum cholesterol and liver cancer (per 1-unit increase)
Figure S11-S18. Forest plots of associations between dietary fats and serum cholesterol and liver cancer (Highest vs. Lowest categories)
Figure S19. Trim-fill methods to adjust publication bias for associations indicating potential publication bias

Table S1 Search terms in current meta-analysis

| Database | Search terms | Items hit |
|---|---|---|
| PubMed | (((hepatocellular OR liver OR hepatic) AND (cancer OR carcinoma OR tumor OR neoplasm)) OR HCC)<br>AND<br>(cholesterol OR fat OR fats OR "fatty acid" OR "fatty acids" OR PUFA OR MUFA OR SFA OR polyunsaturated OR monounsaturated)<br>AND<br>("epidemiological study" OR "epidemiological studies" OR cohort OR longitudinal OR "follow-up" OR prospective OR "case control" OR "cross sectional" OR crosssectional) | 1699 |
| Embase | (((hepatocellular OR liver OR hepatic) AND (cancer OR carcinoma OR tumor OR neoplasm)) OR HCC)<br>AND<br>(cholesterol OR fat OR fats OR "fatty acid" OR "fatty acids" OR PUFA OR MUFA OR SFA OR polyunsaturated OR monounsaturated)<br>AND<br>("epidemiological study" OR "epidemiological studies" OR cohort OR longitudinal OR "follow-up" OR prospective OR "case control" OR "cross sectional" OR crosssectional)<br>in (Title, abstract, key words) | 1428 |
| Web of Science | TOPIC: (((hepatocellular OR liver OR hepatic) AND (cancer OR carcinoma OR tumor OR neoplasm)) OR HCC) AND TOPIC: (cholesterol OR fat OR fats OR "fatty acid" OR "fatty acids" OR PUFA OR MUFA OR SFA OR polyunsaturated OR monounsaturated) AND TOPIC: ("epidemiological study" OR "epidemiological studies" OR cohort OR longitudinal OR "follow-up" OR prospective OR "case control" OR "cross sectional" OR crosssectional) | 1184 |

Table S2 Studies reviewed in full text for eligibility (excluded reasons for meta-analysis)

| No. | Results from full text review | Reasons for excluded |
|---|---|---|
| 1 | Zhang, J., et al. (2019). The alterations of cholesterol, HDL-cholesterol and LDL-cholesterol in Chinese with hepatocellular carcinoma: A cross-sectional study. Asian J Surg 42(10): 938-939. | Letter to editor |
| 2 | Zhang, B., et al. (2017). n-3 fatty acid-based parenteral nutrition improves postoperative recovery for cirrhotic patients with liver cancer: A randomized controlled clinical trial. Clin Nutr 36(5): 1239-1244. | Outcome is not cancer incidence |
| 3 | Wang, C. B., et al. (2015). Fish consumption doesn't reduce the risk of hepatocellular carcinoma. International Journal of Clinical and Experimental Medicine 8(7): 10825-U14211. | Meta-analysis |
| 4 | Venturini, I., et al. (1999). May plasma cholesterol level be considered a neoplastic marker in liver disease from cirrhosis to hepatocellular carcinoma? Ital J Gastroenterol Hepatol 31(1): 61-65. | Only including HCC and cirrhotic control, blood collected from patients |
| 5 | Strohmaier, S., et al. (2013). Total serum cholesterol and cancer incidence in the Metabolic syndrome and Cancer Project (Me-Can). PLoS One 8(1): e54242. | Has been updated |
| 6 | Saito, N., et al. (2013). Low serum LDL cholesterol levels are associated with elevated mortality from liver cancer in Japan: The ibaraki prefectural health study. Tohoku Journal of Experimental Medicine 229(3): 203-211. | Outcome is not cancer incidence |
| 7 | Sahlman, P., et al. (2020). Genetic and lifestyle risk factors for advanced liver disease among men and women. Journal of Gastroenterology and Hepatology (Australia) 35(2): 291-298. | Outcome is liver disease |
| 8 | Reddy, A. V., et al. (2016). Analysis of lipid profile in cancer patients, smokers, and nonsmokers. Dent Res J (Isfahan) 13(6): 494-499. | Without enough data |
| 9 | Radišauskas, R., et al. (2016). Hypertension, serum lipids and cancer risk: A review of epidemiological evidence. Medicina (Kaunas) 52(2): 89-98. | Review article |
| 10 | Otto, C. and W. O. Richter (1995). [Nutrition and cancer. Nutrition-associated risk for the development of various malignancies]. Fortschr Med 113(17): 267-271. | Review article |
| 11 | Nderitu, P., et al. (2016). Association between metabolic syndrome components and the risk of primary liver cancer and cirrhosis. European Journal of Surgical Oncology 42(11): S231. | Abstract |
| 12 | McMichael, A. J., et al. (1984). Dietary and endogenous cholesterol and human cancer. Epidemiol Rev 6: 192-216. | Review article |
| 13 | Mandair, D. S., et al. (2014). The impact of diet and nutrition in the prevention and progression of hepatocellular carcinoma. Expert Review of Gastroenterology & Hepatology 8(4): 369-382. | Review article |
| 14 | Ma, Y. N., et al. (2019). Meat intake and risk of hepatocellular carcinoma in two large US prospective cohorts of women and men. International Journal of Epidemiology 48(6): 1863-1871. | No fat reported |
| 15 | Luo, X., et al. (2019). Type 2 Diabetes Prevention Diet and Hepatocellular Carcinoma Risk in US Men and Women. Am J Gastroenterol 114(12): 1870-1877. | Have been updated |
| 16 | Luo, J., et al. (2014). Systematic review with meta-analysis: meat consumption and the risk of hepatocellular carcinoma. Alimentary Pharmacology & Therapeutics 39(9): 913-922. | Review article |
| 17 | Liu, Y., et al. (2020). Plant-based and animal-based low-carbohydrate diets and risk of hepatocellular carcinoma among US men and women. Hepatology. | Have been updated |
| 18 | Li, W. X. (1993). [Serum cholesterol and cancer mortality: eleven-year prospective cohort study on more than nine thousand persons]. Zhonghua Liu Xing Bing Xue Za Zhi 14(1): 6-9. | Outcome is mortality |
| 19 | Lentjes, M. A. H., et al. (2011). Contribution of cod liver oil supplements to intake and associations with biomarkers of fatty acids in the European Prospective Investigation into Cancer (EPIC-Norfolk) Study. Proceedings of the Nutrition Society 70(OCE3): E103-E103. | Exposure is not of interest |
| 20 | Khattab, M. A., et al. (2012). Association between metabolic abnormalities and hepatitis C-related hepatocellular carcinoma. Ann Hepatol 11(4): 487-494. | No RR reported |
| 21 | Kagan, A., et al. (1981). Serum cholesterol and mortality in a Japanese-American population: the Honolulu Heart program. Am J Epidemiol 114(1): 11-20. | Outcome is not cancer incidence |

| | | |
|---|---|---|
| 22 | Duarte-Salles, T., et al. (2014). Dairy products and risk of hepatocellular carcinoma: the European Prospective Investigation into Cancer and Nutrition. Int J Cancer 135(7): 1662-1672. | Have been updated |
| 23 | Carr, B. I., et al. (2018). Plasma cholesterol and lipoprotein levels in relation to tumor aggressiveness and survival in HCC patients. Int J Biol Markers 33(4): 423-431. | Only have cancer patients |
| 24 | Boada, L. D., et al. (2016). The impact of red and processed meat consumption on cancer and other health outcomes: Epidemiological evidences. Food Chem Toxicol 92: 236-244. | Review article |
| 25 | Biesalski, H. K. (2002). Meat and cancer: meat as a component of a healthy diet. Eur J Clin Nutr 56 Suppl 1: S2-11. | Review article |
| 26 | Bhounsule, P. and A. M. Peterson (2015). Comparison of different metabolic syndrome criteria and individual risk factors in the risk prediction of cardiovascular and chronic diseases. Value in Health 18(3): A135. | Abstract |
| 27 | Abel, S., et al. (2009). Altered lipid profile, oxidative status and hepatitis B virus interactions in human hepatocellular carcinoma. Prostaglandins Leukot Essent Fatty Acids 81(5-6): 391-399. | Without enough data |
| 28 | (2015). Cholesterol and Disease. Annals of Nutrition and Metabolism 66: 14-34. | Review article |
| 29 | Kuper H, Tzonou A, Lagiou P, et al. Diet and hepatocellular carcinoma: a case-control study in Greece. Nutr Cancer. 2000;38(1):6-12. | Without enough data |
| 30 | Wen Y, Wang G, Chen H D, et al. Total cholesterol and the risk of primary liver cancer in Chinese males: a prospective cohort study[J]. Zhonghua yu Fang yi xue za zhi [Chinese Journal of Preventive Medicine], 2020, 54(7): 753-759. | Have been updated |
| 31 | Polesel J, Talamini R, Montella M, et al. Nutrients intake and the risk of hepatocellular carcinoma in Italy[J]. European journal of cancer, 2007, 43(16): 2381-2387. | Case-control study |
| 32 | Hadziyannis S, Tabor E, Kaklamani E, et al. A case-control study of hepatitis B and C virus infections in the etiology of hepatocellular carcinoma[J]. International journal of cancer, 1995, 60(5): 627-631. | Case-control study |

Table S3 Quality assessment of studies included in meta-analysis (Newcastle-Ottawa Quality Assessment Scale)

| No. | First author, year | Study | Selection | | | | | Comparability | | | Outcome | | | | |
|---|---|---|---|---|---|---|---|---|---|---|---|---|---|---|---|
| | | | Q1 | Q2 | Q3 | Q4 | Sub-Total | Q5 A | Q5 B | Sub-Total | Q6 | Q7 | Q8 | Sub-Total | Total |
| 1 | Li, 2020 | 4C Cohort | 1 | 1 | 1 | 1 | 4 | 1 | 1 | 2 | 1 | 0 | 1 | 2 | 8 |
| 2 | Yang, 2020 | NHS/HPFS | 0 | 1 | 1 | 1 | 3 | 1 | 1 | 2 | 1 | 1 | 1 | 3 | 8 |
| 3 | Nderitu, 2017 | Swedish AMORIS | 1 | 1 | 1 | 1 | 4 | 1 | 0 | 1 | 1 | 1 | 0 | 2 | 7 |
| 4 | Guan, 2017 | Kailuan Cohort | 0 | 1 | 1 | 1 | 3 | 1 | 1 | 2 | 1 | 1 | 0 | 2 | 7 |
| 5 | Koh, 2016 | SCHS | 1 | 1 | 1 | 1 | 4 | 1 | 1 | 2 | 1 | 1 | 1 | 3 | 9 |
| 6 | Duarte-Salles, 2015 | EPIC | 1 | 1 | 1 | 1 | 4 | 1 | 1 | 2 | 1 | 1 | 1 | 3 | 9 |
| 7 | Sawada, 2012 | JPHC | 1 | 1 | 1 | 1 | 4 | 1 | 1 | 2 | 1 | 1 | 1 | 3 | 9 |
| 8 | Borena, 2011 | Me-Can | 1 | 1 | 1 | 1 | 4 | 1 | 1 | 2 | 1 | 1 | 0 | 2 | 8 |
| 9 | Kitahara, 2011 | KCPS | 1 | 1 | 1 | 1 | 4 | 1 | 1 | 2 | 1 | 1 | 1 | 3 | 9 |
| 10 | Freedman, 2010 | NIH-AARP | 1 | 1 | 1 | 1 | 4 | 1 | 1 | 2 | 1 | 0 | 0 | 1 | 7 |
| 11 | Ahn, 2009 | ATBC | 1 | 1 | 1 | 1 | 4 | 1 | 1 | 2 | 1 | 1 | 1 | 3 | 9 |
| 12 | Iso, 2009 | JPHC | 1 | 1 | 1 | 1 | 4 | 1 | 1 | 2 | 1 | 1 | 1 | 3 | 9 |
| 13 | Ioannou, 2009 | NHANES | 1 | 1 | 0 | 1 | 3 | 1 | 1 | 2 | 0 | 1 | 0 | 1 | 6 |
| 14 | Strasak, 2009 | VHM&PP | 1 | 1 | 1 | 1 | 4 | 1 | 1 | 2 | 1 | 1 | 0 | 2 | 8 |

Abbreviations: 4C, the China Cardiometabolic Disease and Cancer Cohort Study; AMORIS, The Swedish Apolipoprotein Mortality Risk Study; ATBC, the Alpha-Tocopherol, Beta-Carotene Cancer Prevention Study cohort; EPIC, the European Prospective Investigation into Cancer and Nutrition cohort; HPFS, the Health Professional Follow-up Study; JPHC, the Japan Public Health Center-based prospective study; KCPS, the Korean Cancer Prevention Study; NHANES, the National Health and Nutrition Examination Survey; NHS, the Nurse Health Study; SCHS, the Singapore Chinese Health Study; SES, social economic status; VHM&PP, the Vorarlberg Health Monitoring and Promotion Program.

Q5 A means whether study controlled for age and sex; Q5 B means whether study controlled for at least two of the following potential confounders: family history, BMI, energy intake, smoke, alcohol.

Table S4 Sensitivity analyses of associations between dietary fat, serum cholesterol and liver cancer ( number of studies ≥ 6)

| Exposure and study | RR (95%CI) | P | $I^2$ | P for Q |
|---|---|---|---|---|
| **Serum cholesterol (per 1 mmol/L)** | | | | |
| Borena, 2011, Both | 0.72 (0.69, 0.76) | < 0.001 | < 0.001 | 77.881 |
| Ahn, 2009, M | 0.71 (0.68, 0.75) | < 0.001 | 0.001 | 76.282 |
| Nderitu, 2017, Both | 0.72 (0.68, 0.76) | < 0.001 | < 0.001 | 78.91 |
| Kitahara, 2011, M | 0.68 (0.61, 0.76) | < 0.001 | < 0.001 | 78.053 |
| Kitahara, 2011, F | 0.68 (0.61, 0.76) | < 0.001 | < 0.001 | 79.127 |
| Iso, 2009, M | 0.73 (0.71, 0.75) | < 0.001 | 0.072 | 50.642 |
| Iso, 2009, F | 0.72 (0.69, 0.76) | < 0.001 | 0.001 | 75.662 |
| | | | | |
| **Serum cholesterol (H/L)** | | | | |
| Borena, 2011, Both | 0.38 (0.31, 0.47) | < 0.001 | 0.002 | 71.49 |
| Kitahara, 2011, M | 0.34 (0.25, 0.45) | < 0.001 | 0.001 | 72.44 |
| Kitahara, 2011, F | 0.37 (0.29, 0.47) | < 0.001 | 0.005 | 67.47 |
| Ahn, 2009, M | 0.34 (0.28, 0.42) | < 0.001 | 0.003 | 69.82 |
| Nderitu, 2017, Both | 0.34 (0.26, 0.44) | < 0.001 | 0.001 | 74.5 |
| Guan, 2017, M | 0.38 (0.31, 0.47) | < 0.001 | 0.001 | 72.6 |
| Iso, 2009, M | 0.37 (0.30, 0.46) | < 0.001 | 0.001 | 74.15 |
| Iso, 2009, F | 0.37 (0.30, 0.46) | < 0.001 | 0.001 | 74.02 |

SF1. Dietary fat and HCC

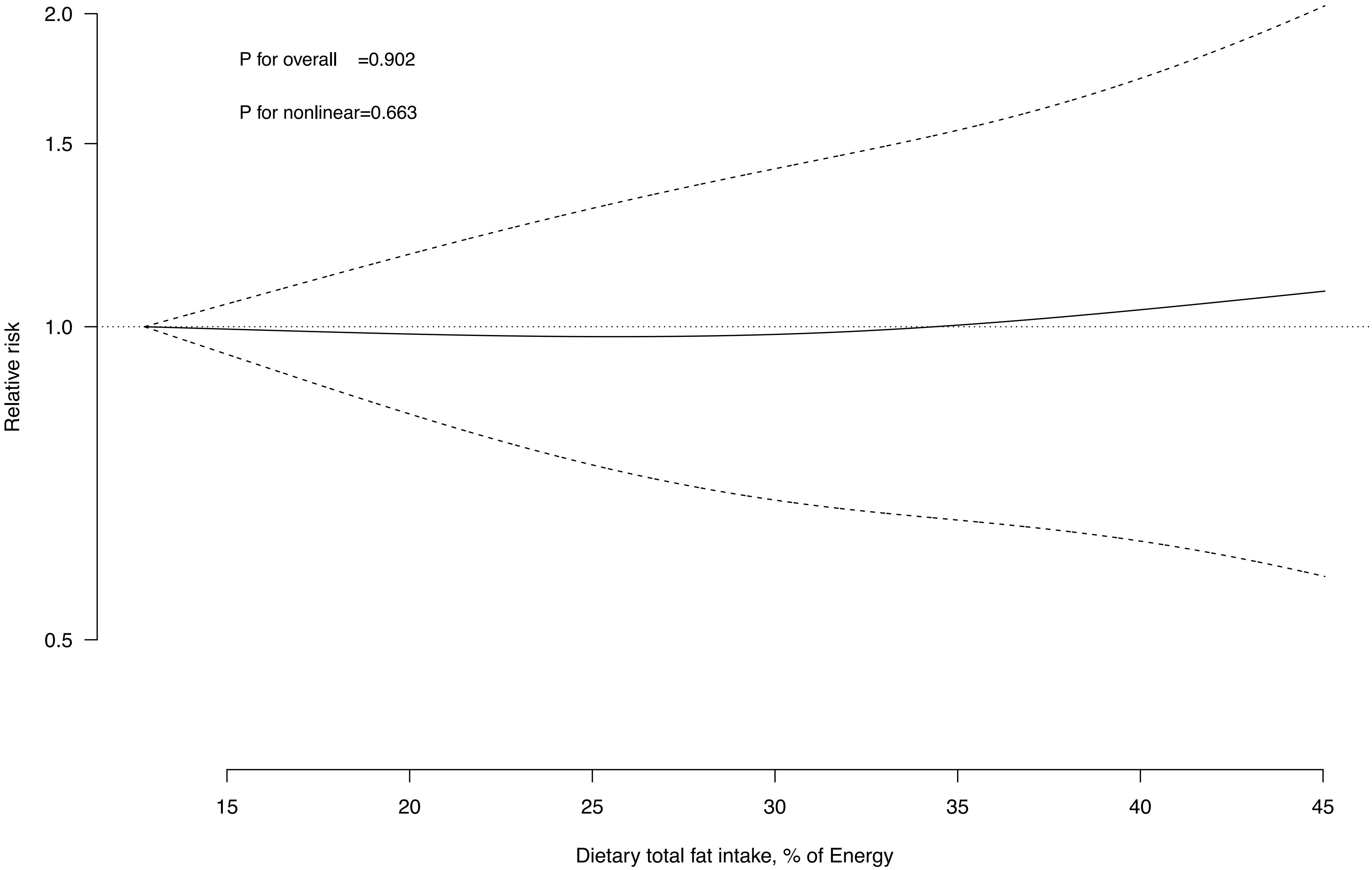

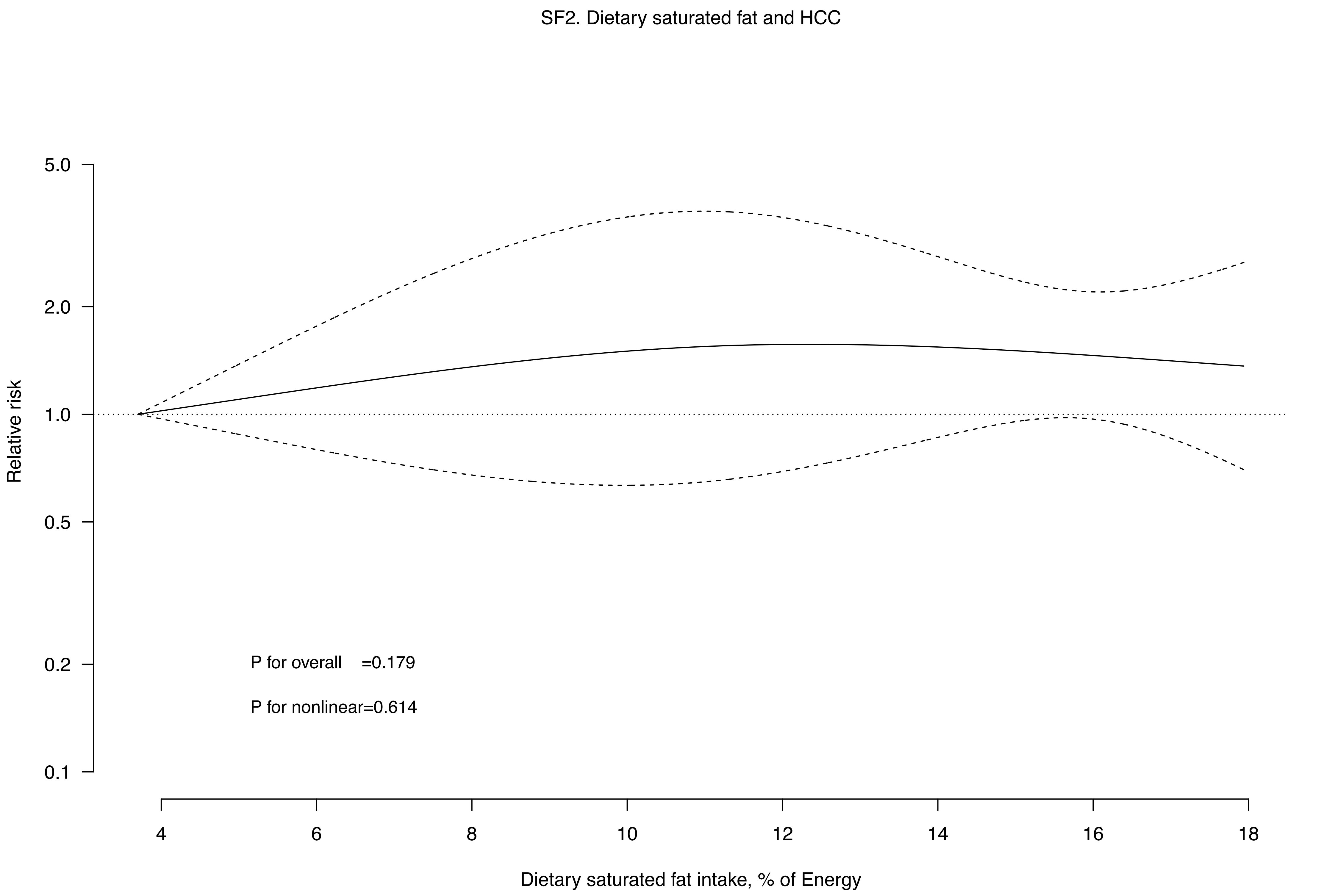
SF2. Dietary saturated fat and HCC
5.0
2.0
1.0
0.5
0.2
0.1
Relative risk
P for overall =0.179
P for nonlinear=0.614
4
6
8
10
12
14
16
18
Dietary saturated fat intake, % of Energy

SF3. Serum cholestrol and HCC

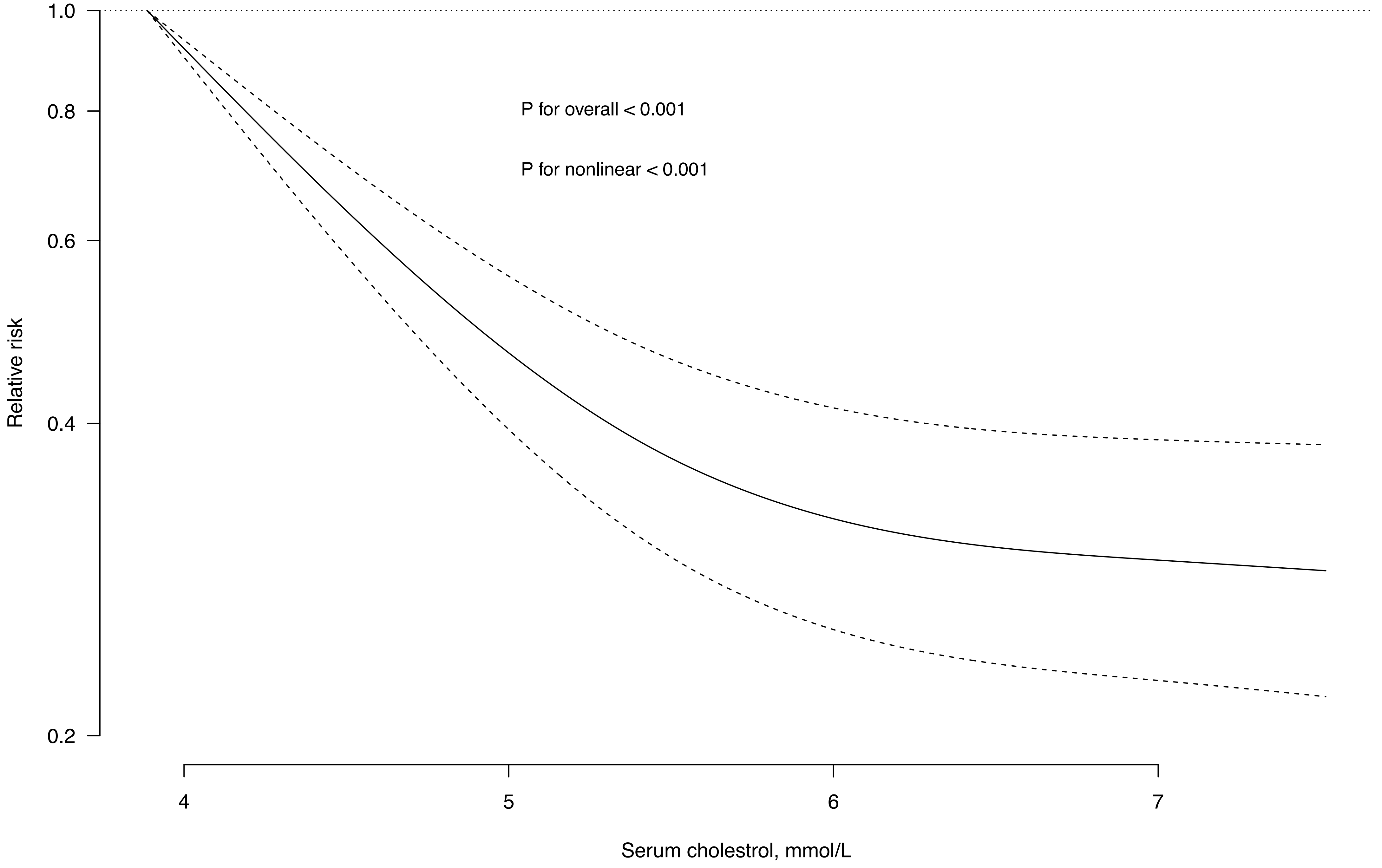

SF4. Cholesterol in serum, per mmol/L

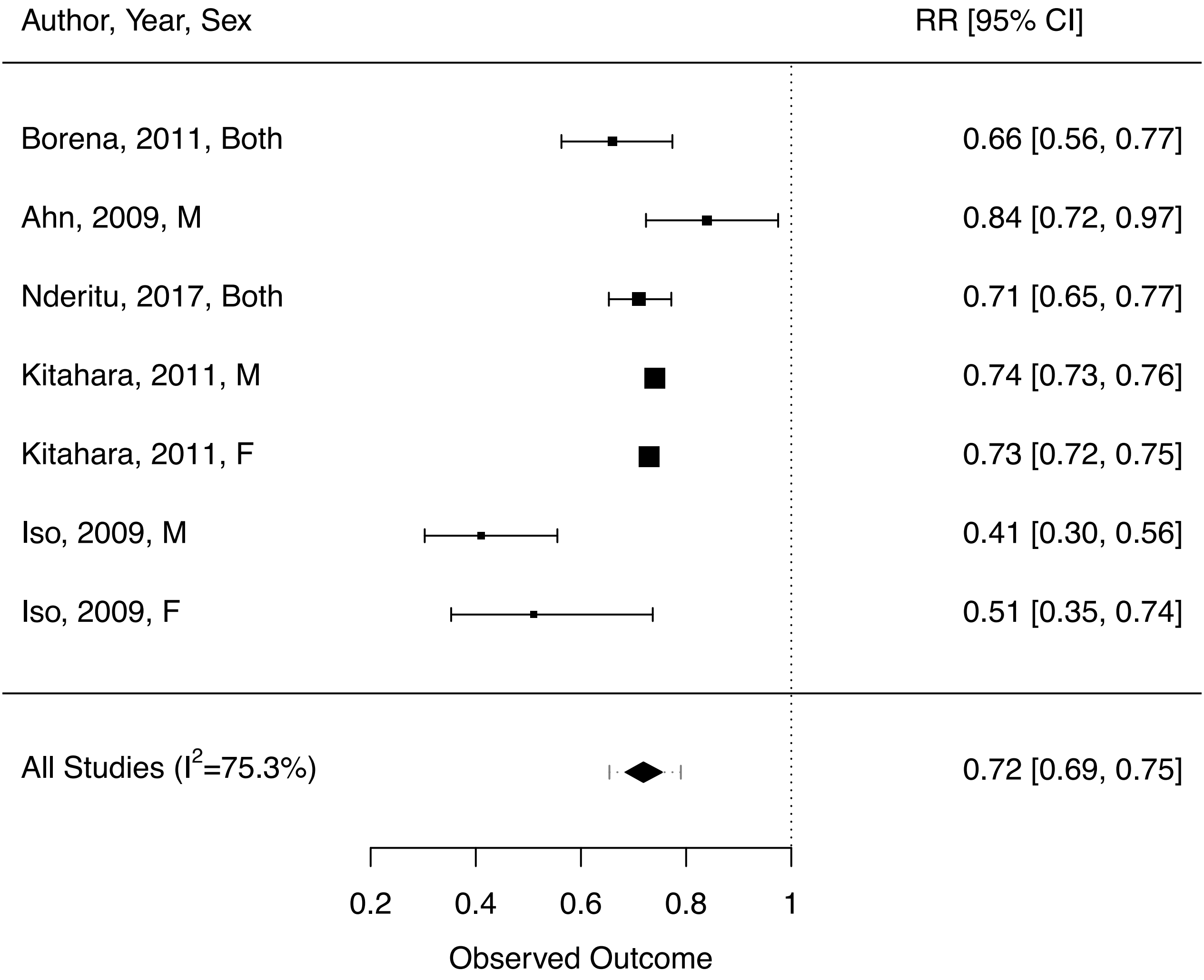

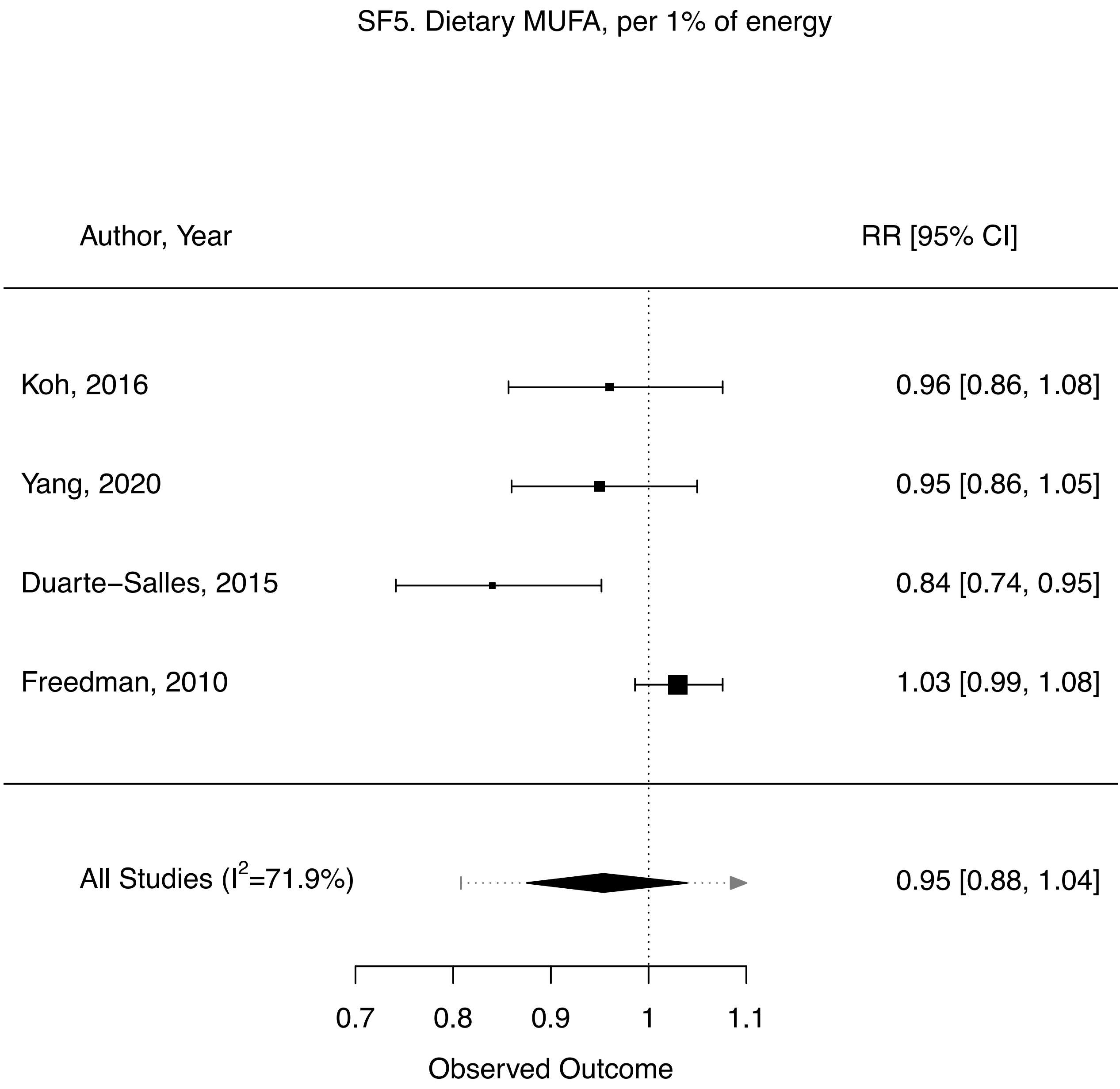
SF5. Dietary MUFA, per 1% of energy
Author, Year
RR [95% CI]
Koh, 2016
0.96 [0.86, 1.08]
Yang, 2020
0.95 [0.86, 1.05]
Duarte−Salles, 2015
0.84 [0.74, 0.95]
Freedman, 2010
1.03 [0.99, 1.08]
All Studies (I²=71.9%)
0.95 [0.88, 1.04]
0.7
0.8
0.9
1
1.1
Observed Outcome

SF6. Dietary N3 PUFA, per 1% of energy

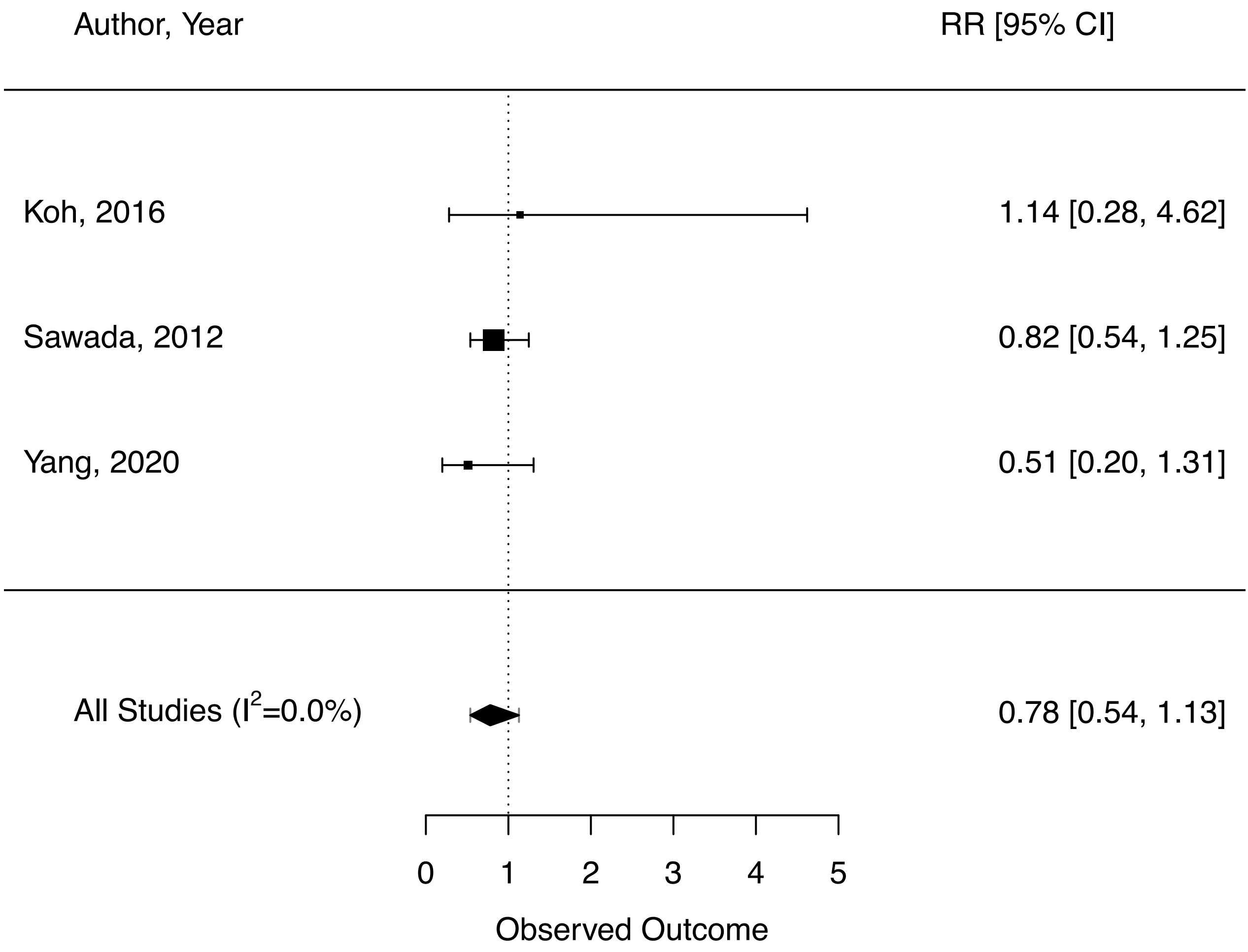

SF7. Dietary PUFA, per 1% of energy

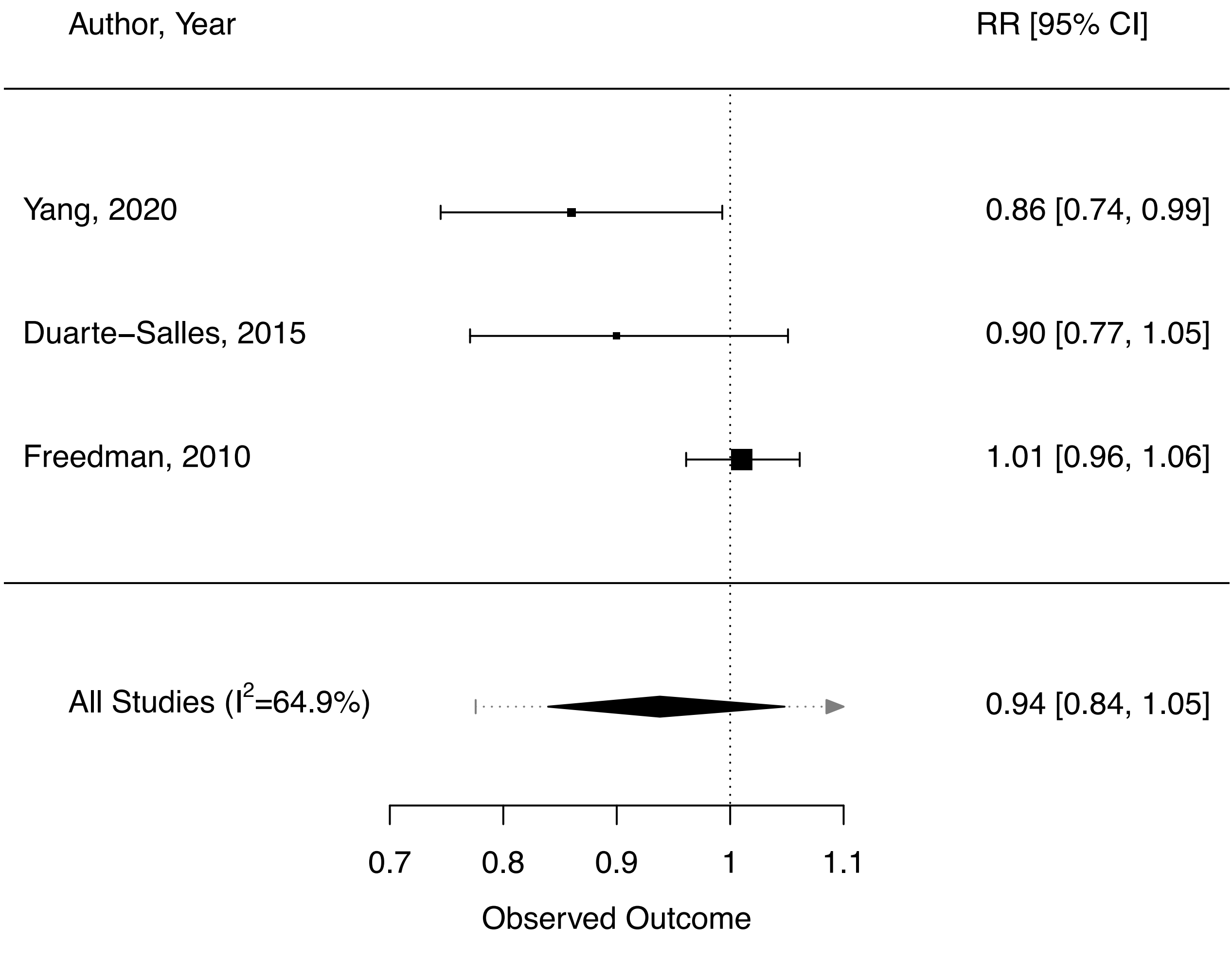

SF8. Dietary saturated fat, per 1% of energy

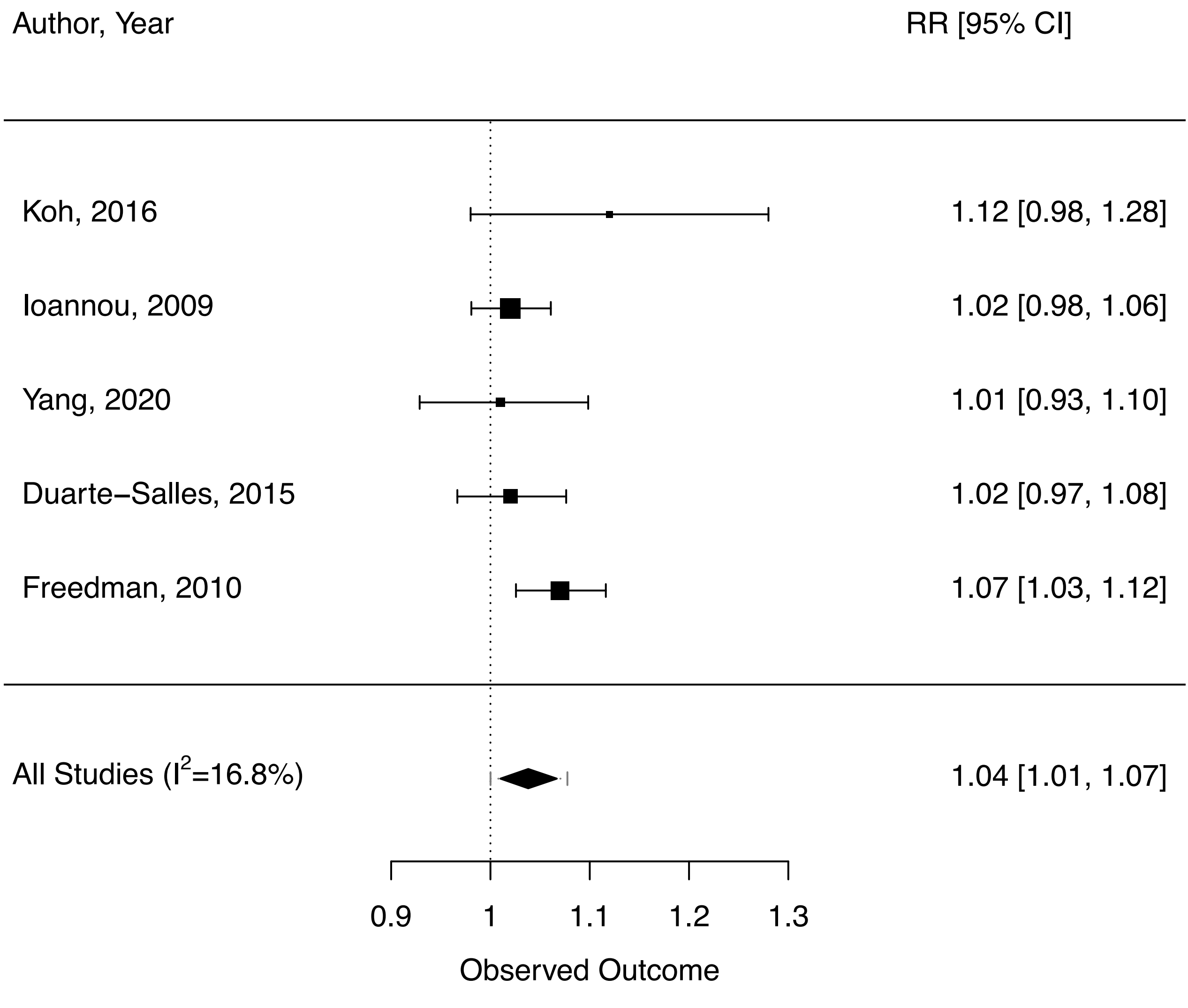

SF9. Dietary total fat, per 5% of energy

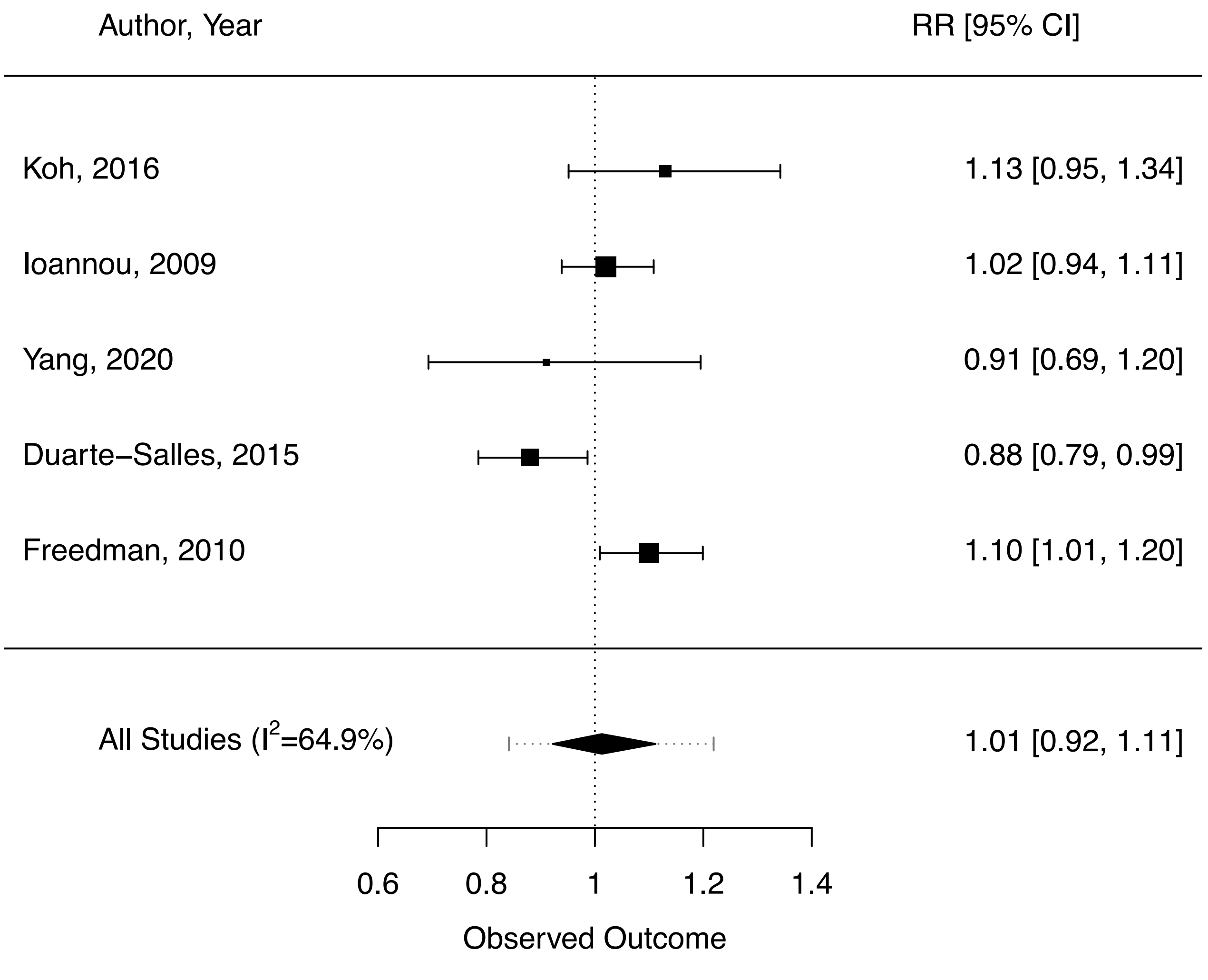

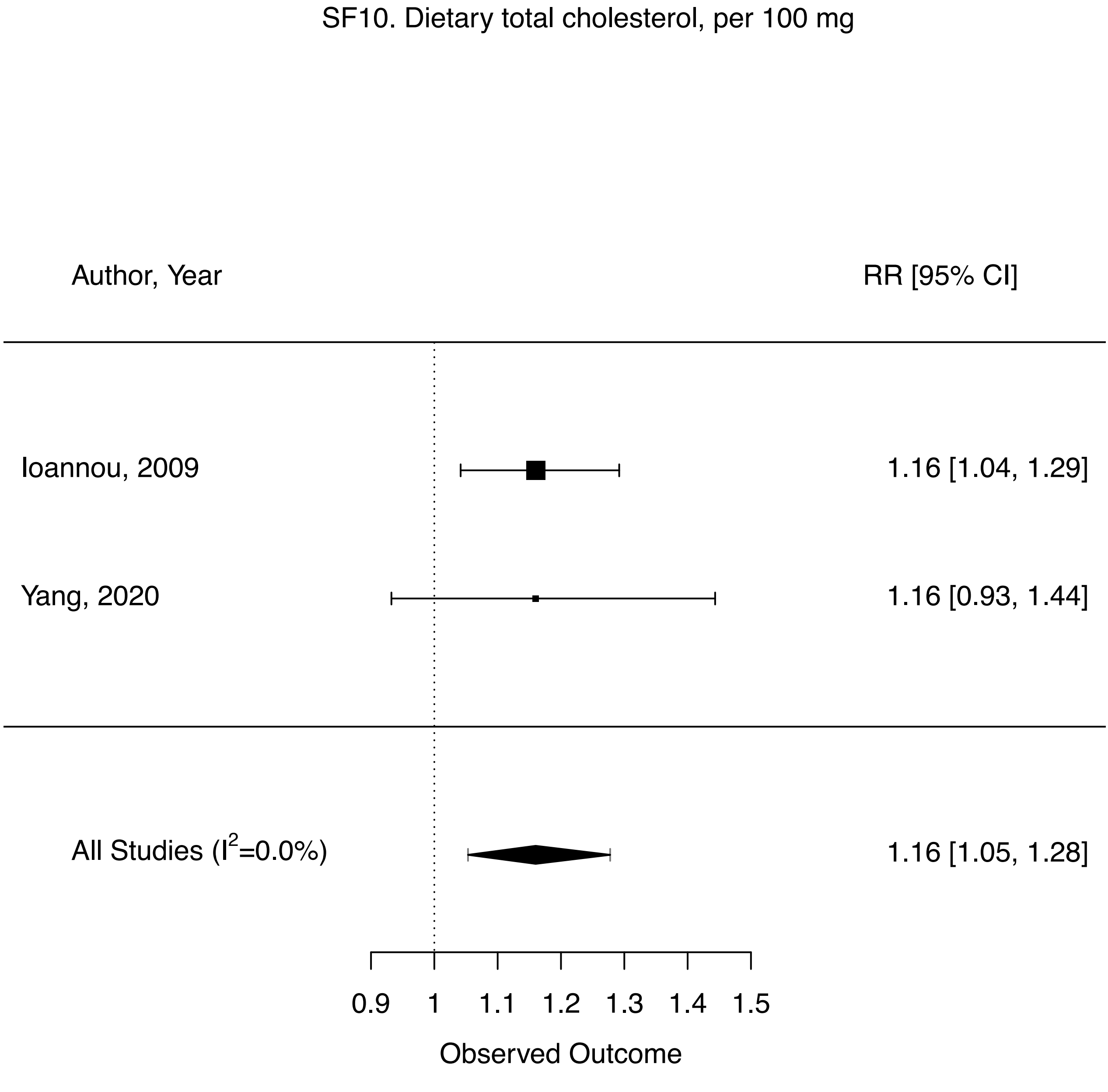
SF10. Dietary total cholesterol, per 100 mg
Author, Year
RR [95% CI]
Ioannou, 2009
1.16 [1.04, 1.29]
Yang, 2020
1.16 [0.93, 1.44]
All Studies (I2=0.0%)
1.16 [1.05, 1.28]
0.9
1
1.1
1.2
1.3
1.4
1.5
Observed Outcome

SF11. Cholesterol in serum, H/L (4 subgroups for Strasak,2009)

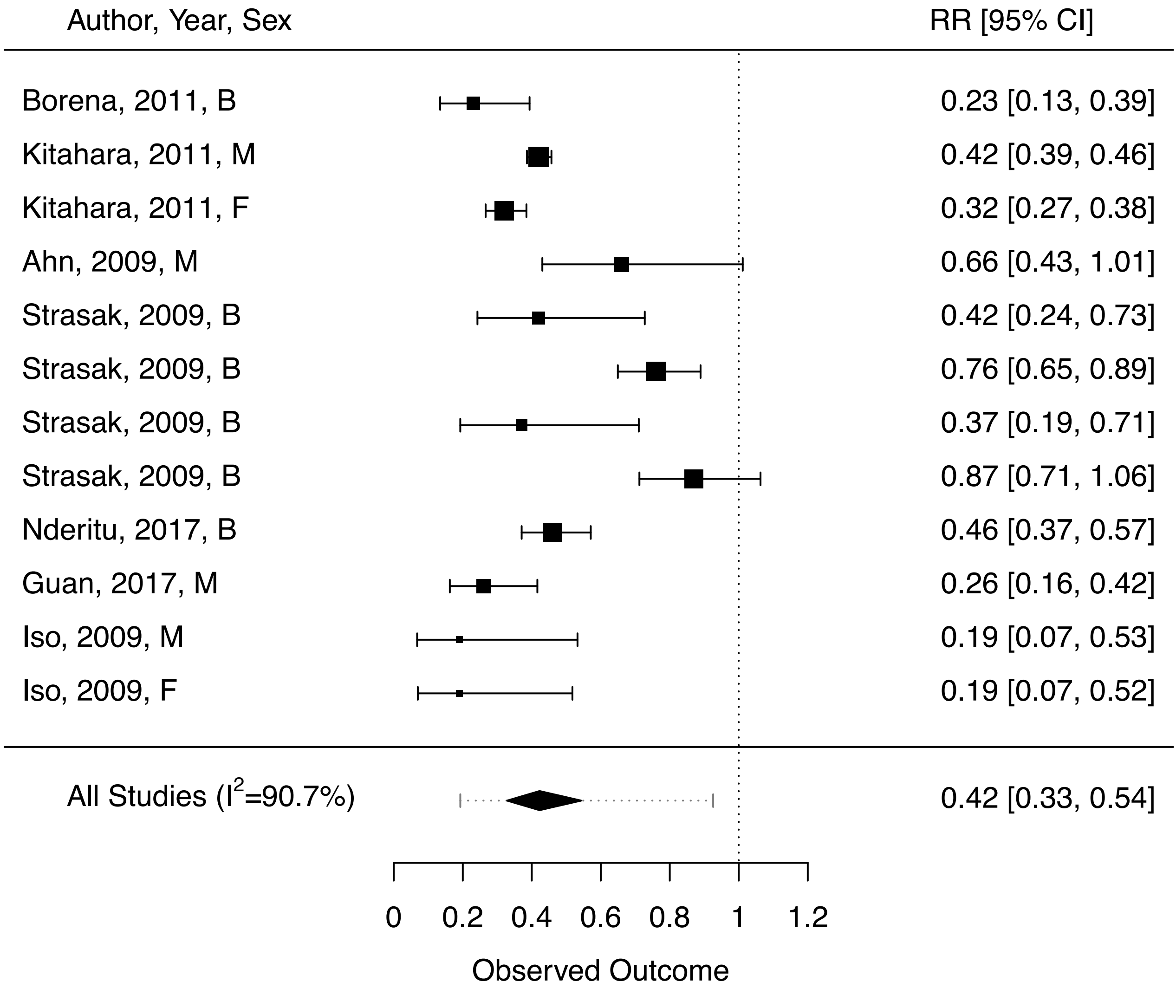

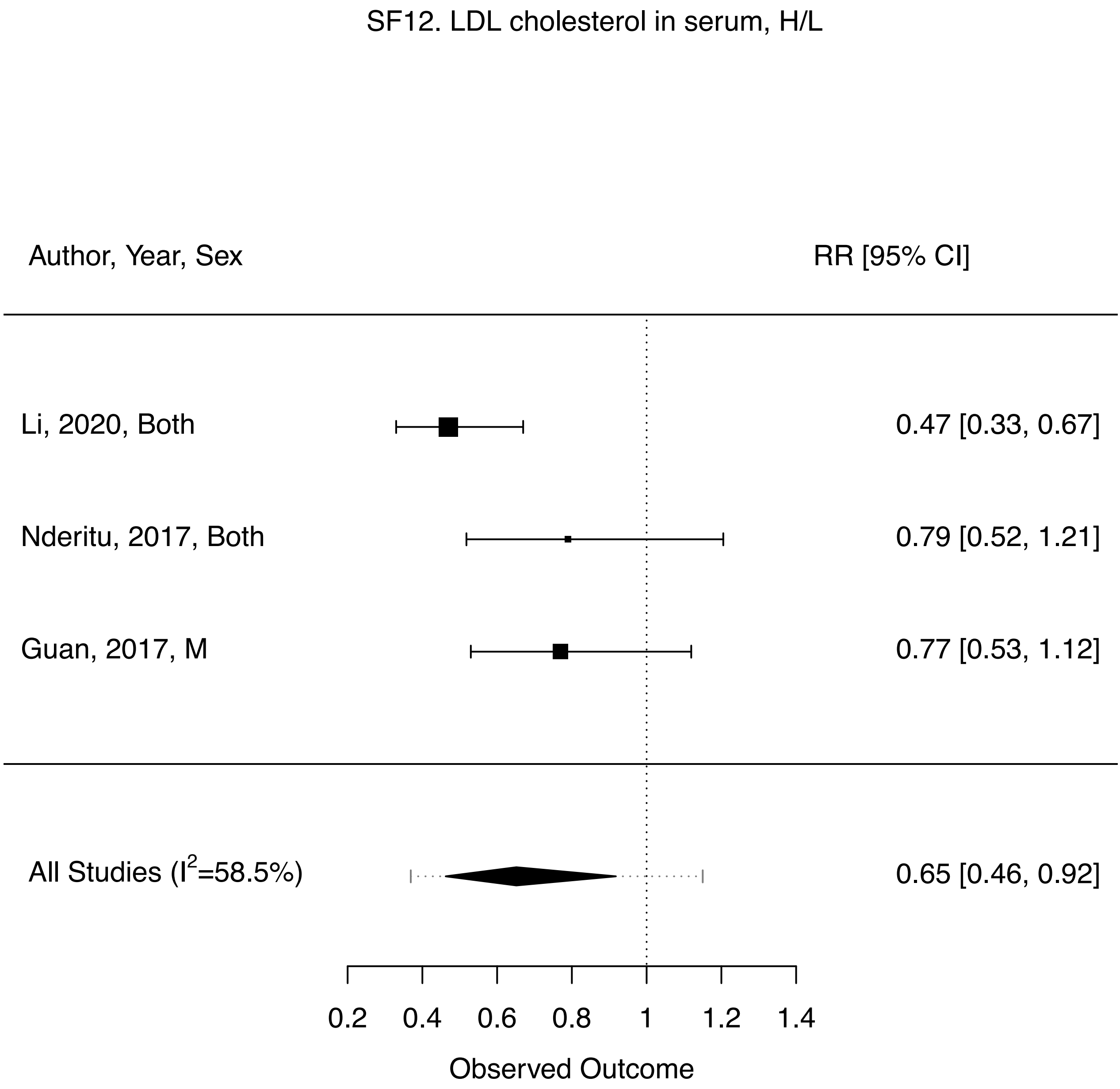
SF12. LDL cholesterol in serum, H/L
Author, Year, Sex
RR [95% CI]
Li, 2020, Both
0.47 [0.33, 0.67]
Nderitu, 2017, Both
0.79 [0.52, 1.21]
Guan, 2017, M
0.77 [0.53, 1.12]
All Studies (I2=58.5%)
0.65 [0.46, 0.92]
0.2
0.4
0.6
0.8
1
1.2
1.4
Observed Outcome

SF13. Cholesterol in diet, H/L

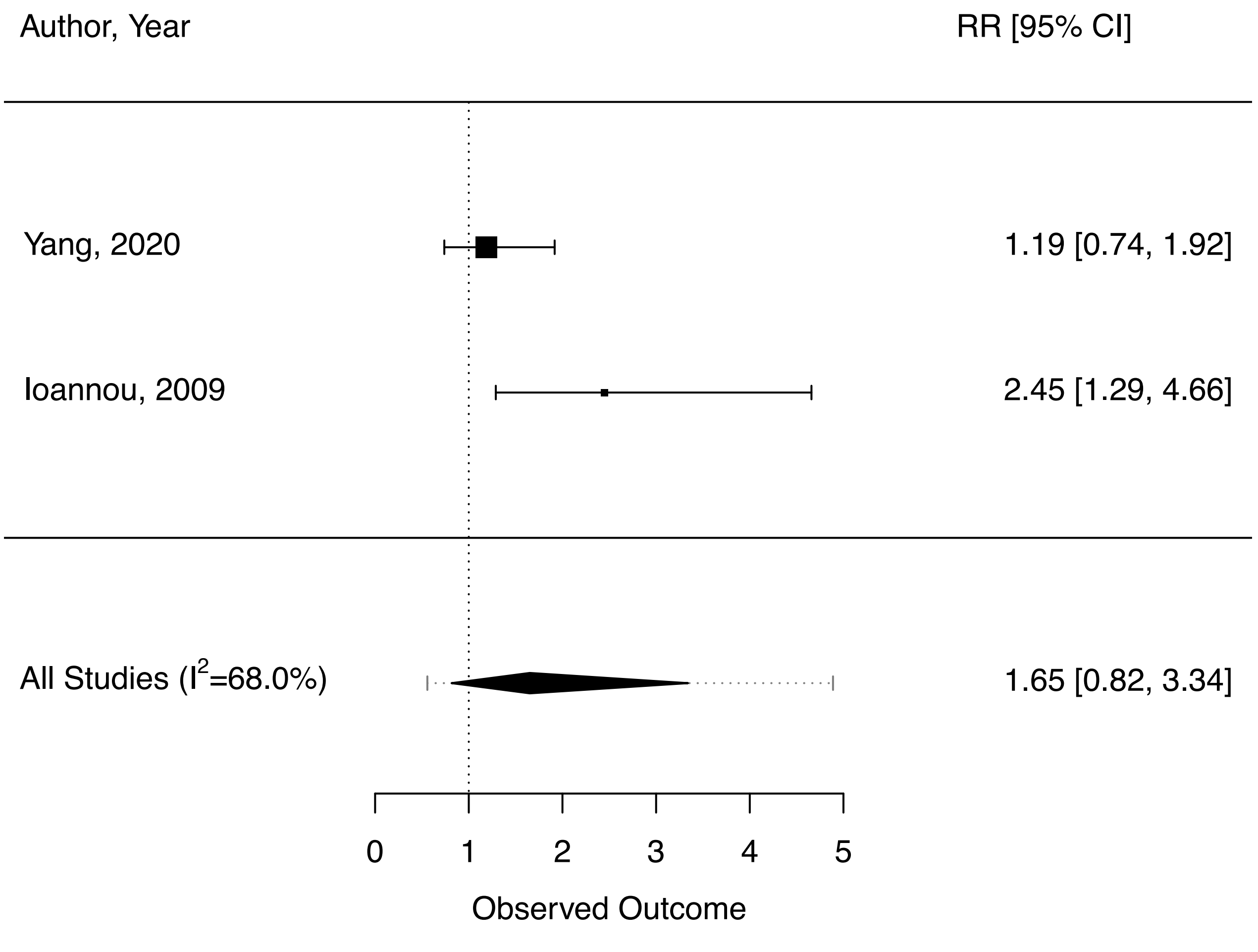

SF14. Dietary MUFA, H/L

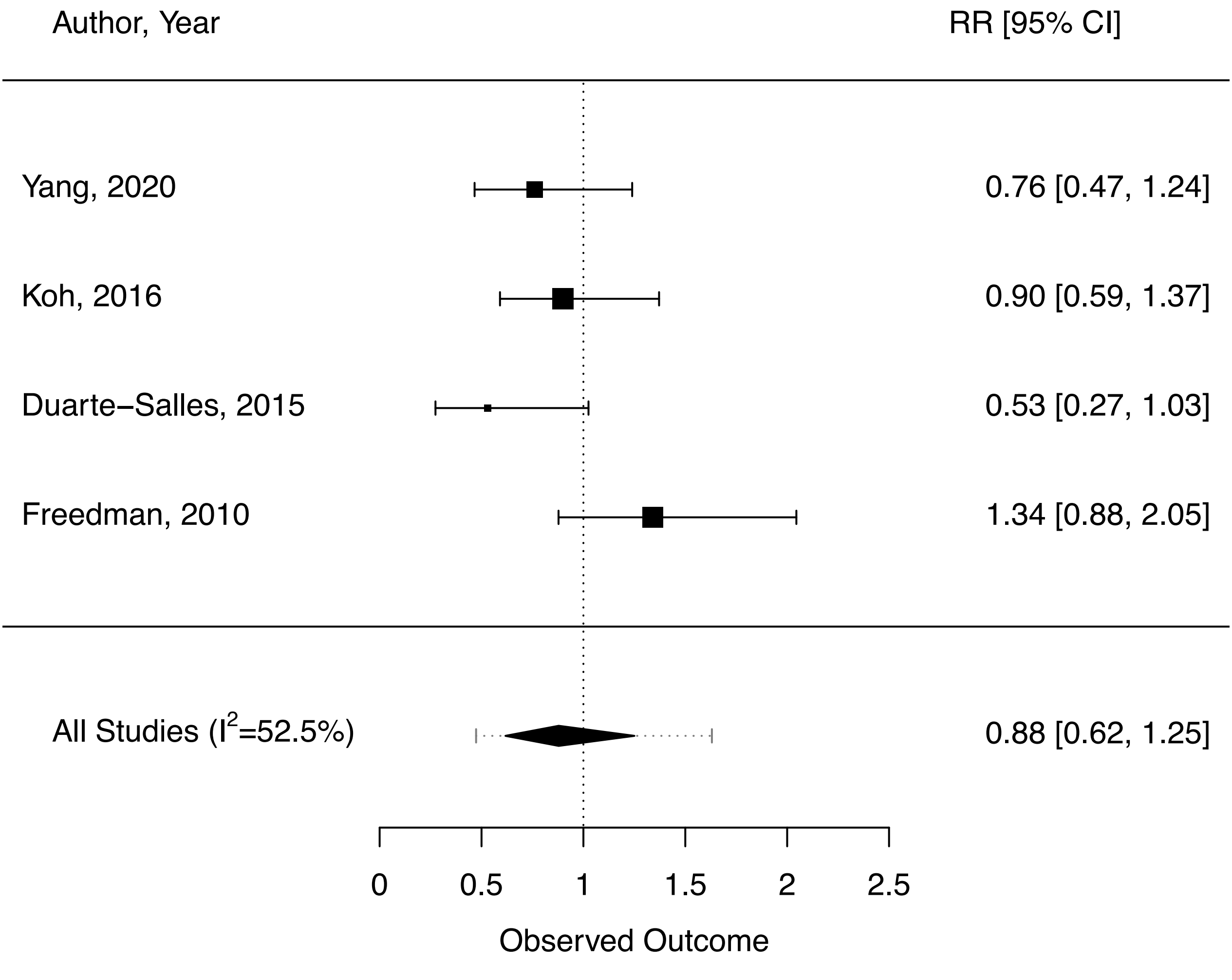

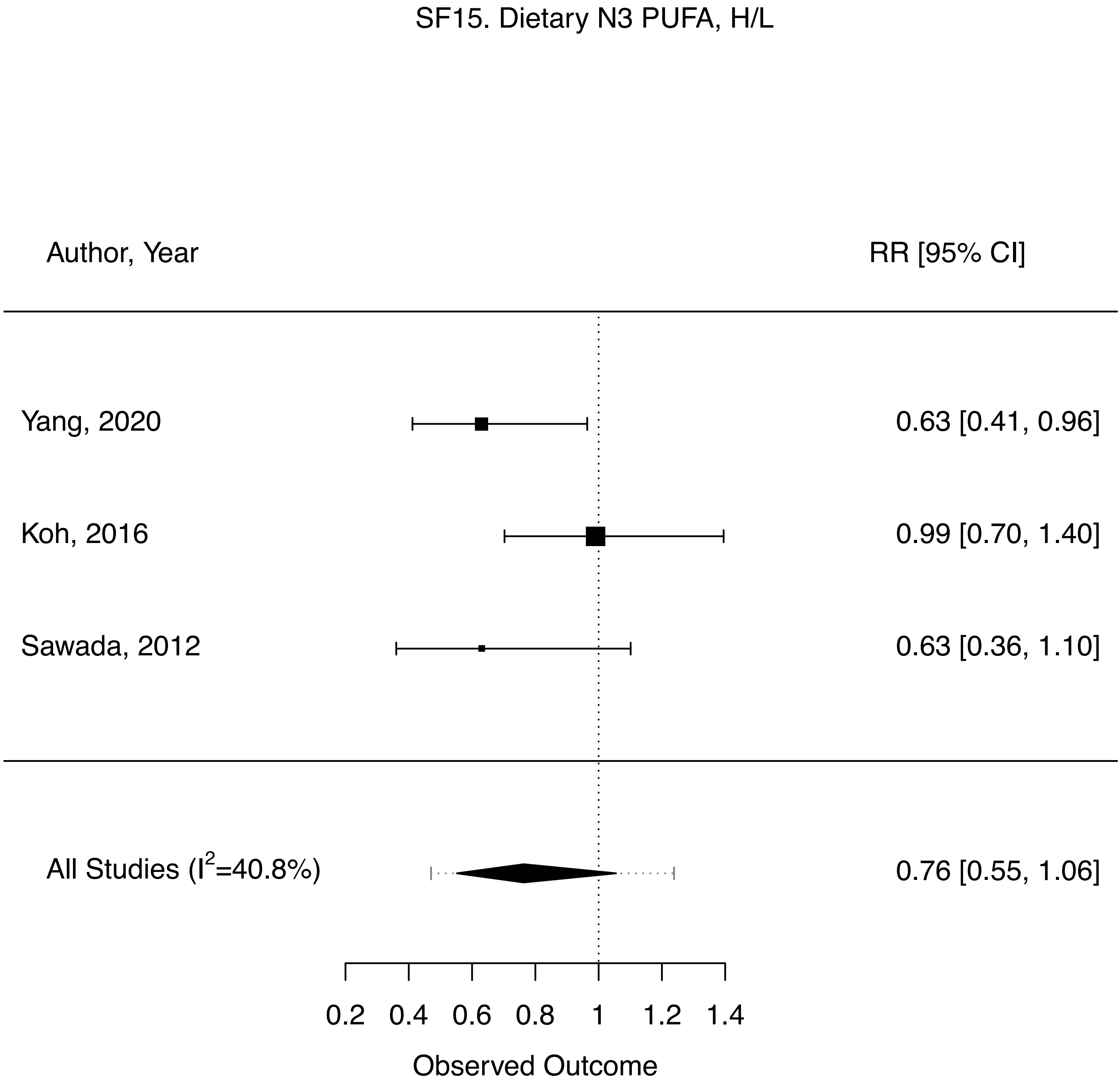
SF15. Dietary N3 PUFA, H/L
Author, Year
RR [95% CI]
Yang, 2020
0.63 [0.41, 0.96]
Koh, 2016
0.99 [0.70, 1.40]
Sawada, 2012
0.63 [0.36, 1.10]
All Studies (I2=40.8%)
0.76 [0.55, 1.06]
0.2
0.4
0.6
0.8
1
1.2
1.4
Observed Outcome

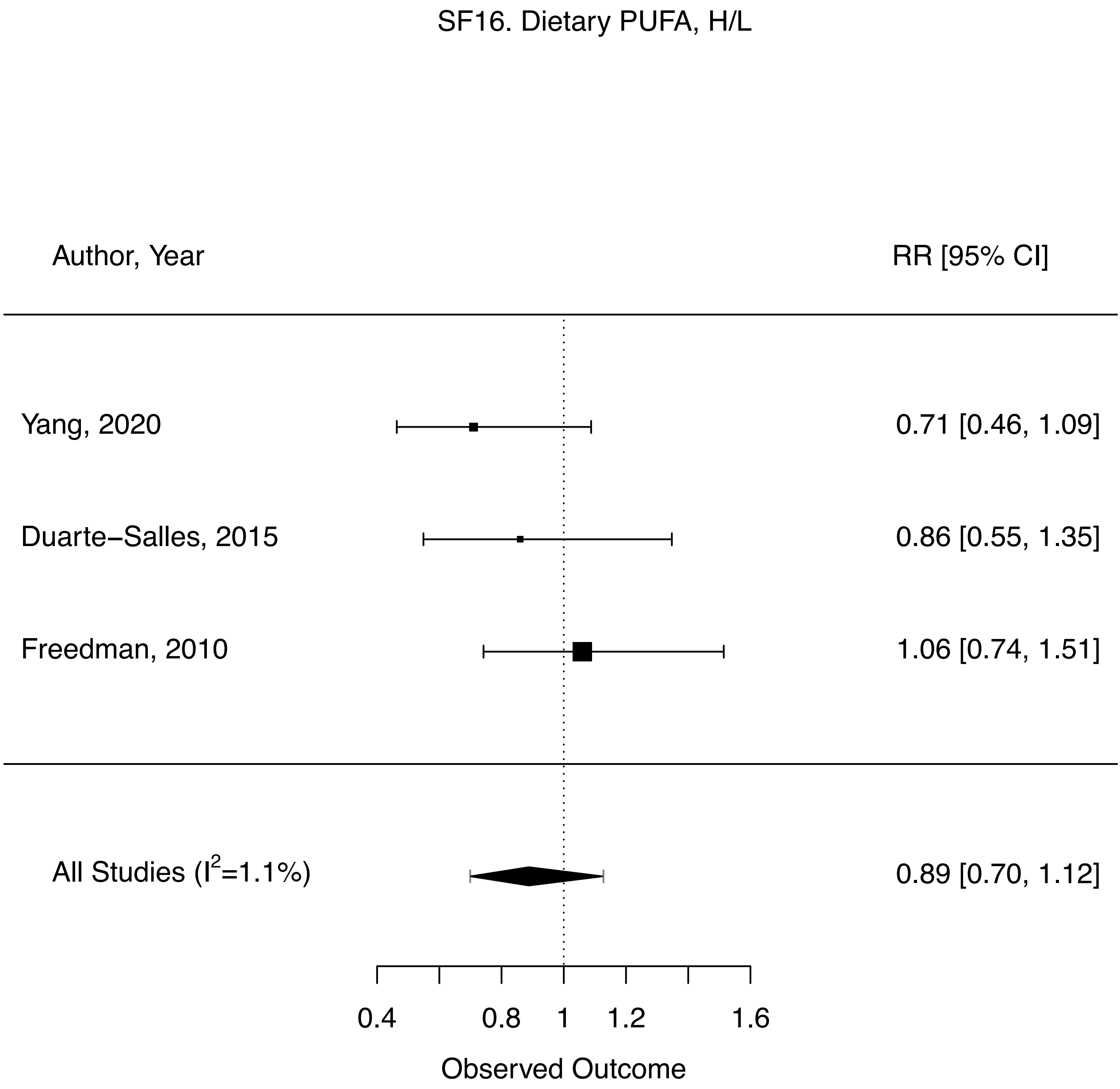
SF16. Dietary PUFA, H/L
Author, Year
RR [95% CI]
Yang, 2020
0.71 [0.46, 1.09]
Duarte−Salles, 2015
0.86 [0.55, 1.35]
Freedman, 2010
1.06 [0.74, 1.51]
All Studies ($I^2$=1.1%)
0.89 [0.70, 1.12]
0.4
0.8
1
1.2
1.6
Observed Outcome

SF17. Dietary saturated fat, H/L

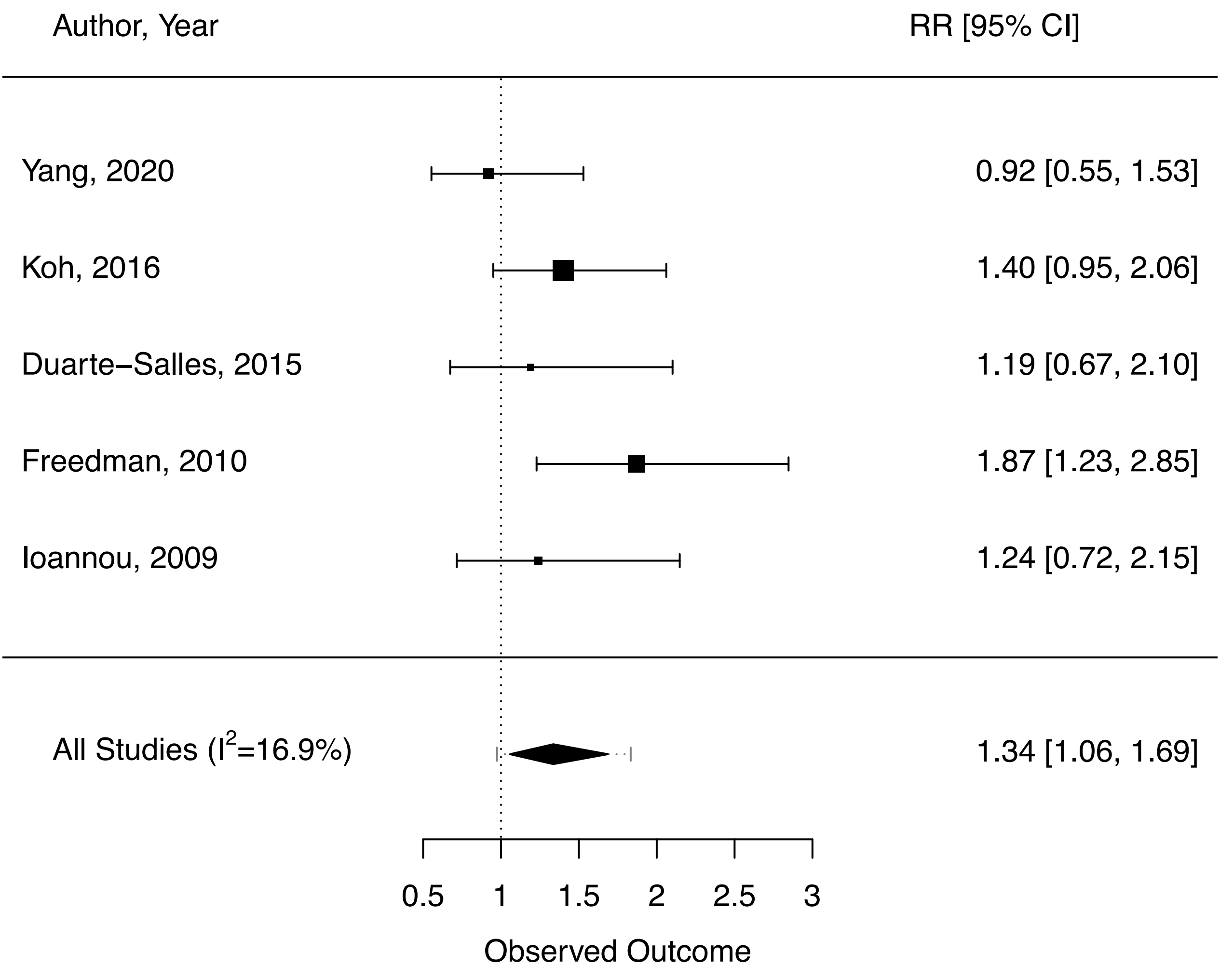

SF18. Dietary total fat, H/L

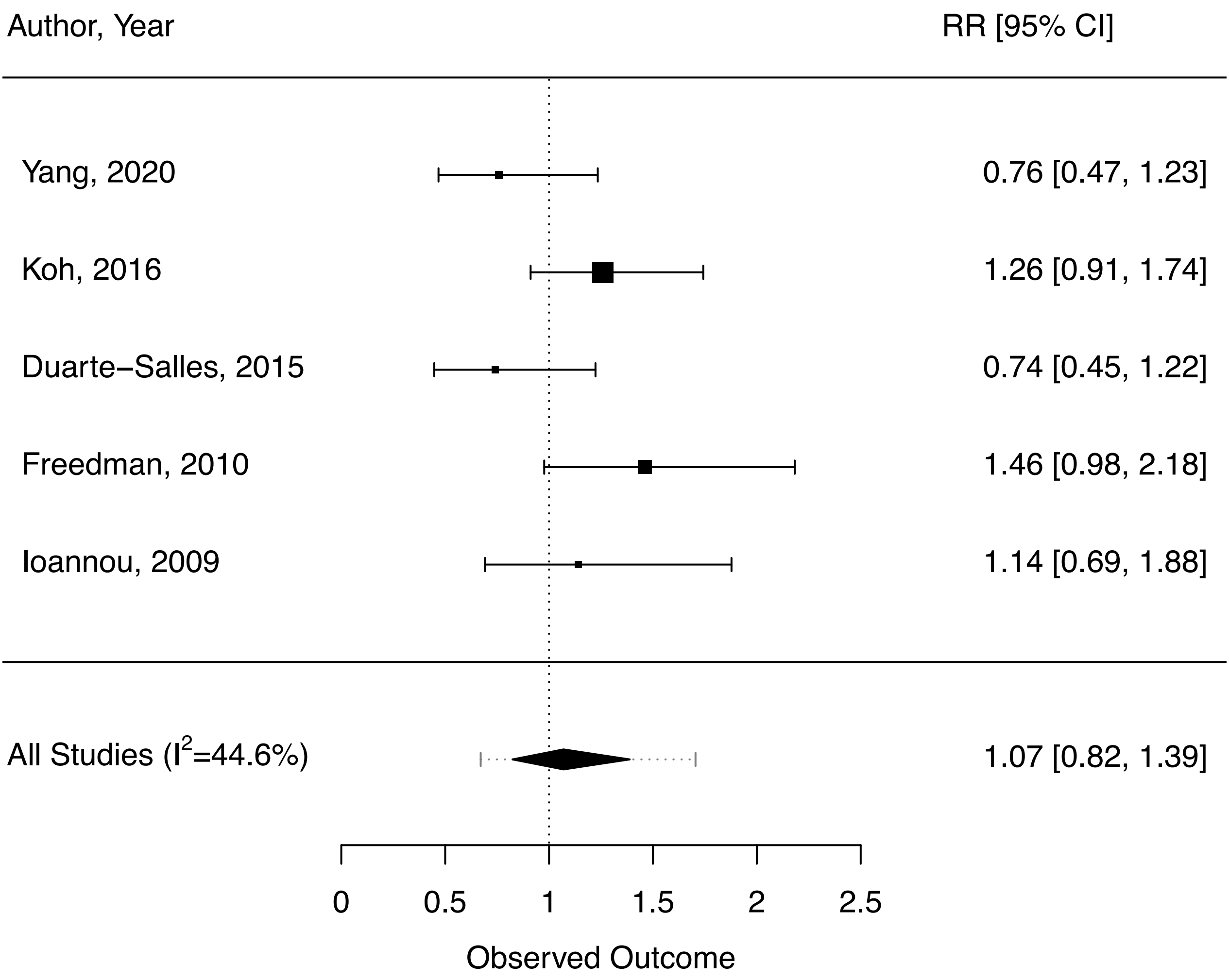

**Supplemental figure 19. Trim-fill methods to adjust publication bias**

a. Total cholesterol in serum (per 1 mmol/L)

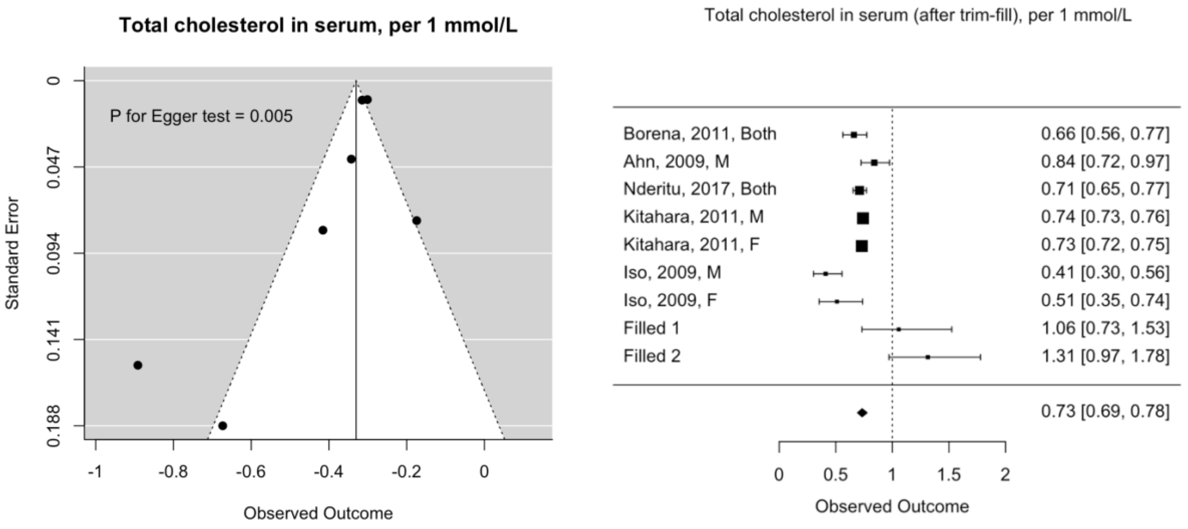


b. Total cholesterol in serum (H/L)

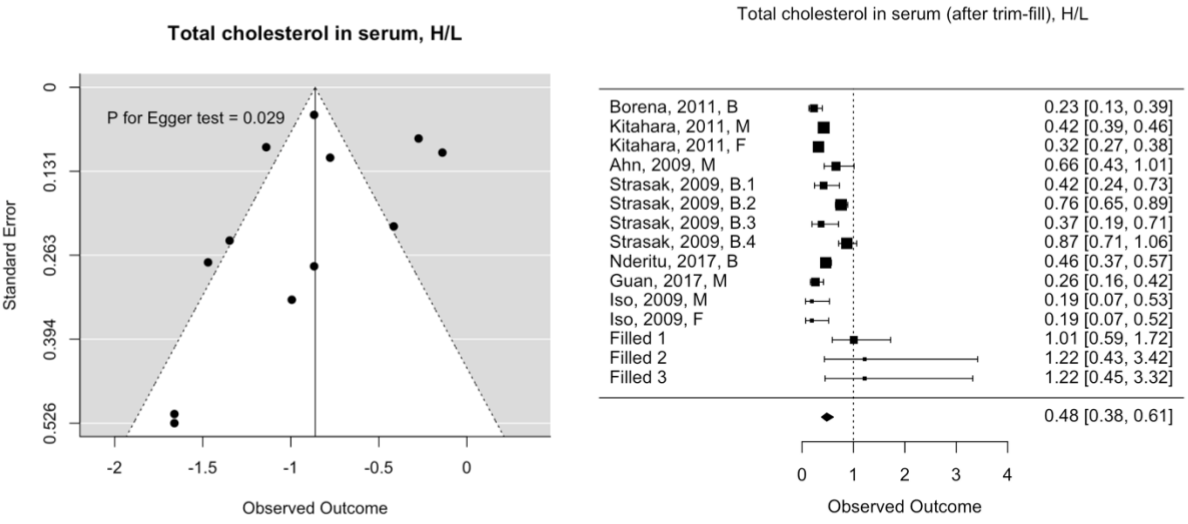

Supplement: Supplementary file 1 [file cancers-13-01580-s001.pdf]
